# Burden of *Shigella* among children with diarrhea in the Americas: A systematic review and meta-analysis

**DOI:** 10.1371/journal.pntd.0013393

**Published:** 2025-08-18

**Authors:** Maya Lubeck-Schricker, Andrea C. Rivas-Nieto, Jennifer Rosauer, Samuel Mpinganjira, Akash Malhotra, Magdalena Bastias, Elizabeth Rogawski McQuade, Margaret Kosek, Claudio F. Lanata, Maribel Paredes Olortegui, Theresa J. Ochoa, James A. Platts-Mills, Kirsten Vannice, Patricia B. Pavlinac

**Affiliations:** 1 Department of Epidemiology, University of Washington, Seattle, Washington, United States of America; 2 Department of Global Health, University of Washington, Seattle, Washington, United States of America; 3 Pan-American Health Organization, Washington, DC, United States of America; 4 Department of Epidemiology, Emory University, Atlanta, Georgia, United States of America; 5 Asociación Benéfica PRISMA, Iquitos, Peru; 6 Division of Infectious Diseases and International Health, University of Virginia, Charlottesville, Virginia, United States of America; 7 Instituto de Investigación Nutricional, Lima, Peru; 8 Department of Pediatrics, Vanderbilt University, Nashville, Tennessee, United States of America; 9 Instituto de Medicina Tropical Alexander von Humboldt, Universidad Peruana Cayetano Heredia, Lima, Peru; 10 The Gates Foundation, Seattle, Washington, United States of America; University of Oxford, UNITED KINGDOM OF GREAT BRITAIN AND NORTHERN IRELAND

## Abstract

**Introduction:**

*Shigella* is a leading cause of diarrhea worldwide. While the burden of *Shigella* has been shown to be highest in Africa and Asia, recent studies have also shown considerable burden in the Americas. With several pediatric *Shigella* vaccines in clinical development, policymakers in the region will eventually consider whether a *Shigella* vaccine is appropriate for their setting.

**Methods:**

We conducted a systematic review and meta-analyses to summarize the burden (characterized by prevalence, incidence, and attributable fraction estimates) of *Shigella* diarrhea among children under 72 months in the Americas, excluding the U.S., Canada, and Greenland. We searched published and pre-print articles available in six databases from January 1, 2000 through July 18, 2024. Random effects meta-analyses were conducted for subgroups of interest when relevant data from at least two studies were present.

**Results:**

This review included 34 studies conducted across 14 countries in the region. Prevalence was most frequently reported, followed by incidence, then attributable fraction. Across all prevalence studies that used a culture detection method (n = 23), the pooled prevalence of *Shigella* among diarrhea cases was 3.1% (95% CI: 1.6- 5.8). The pooled prevalence among 7 studies that used PCR/qPCR detection methods was 16.5% (95% CI: 11.1-24.0). Among culture-based results, the pooled prevalence estimate for children <12 months was 1.0% (95% CI: 0.1 – 7.7) compared to 4.6% (95% CI: 1.2 – 15.4) for children ≥12 months.

**Conclusion:**

Despite varying reporting practices, we found *Shigella* to be an important contributor to diarrhea in many settings in the Americas with substantial heterogeneity. Limited geographic representation and variable reporting of age group specific estimates were the major gaps in data. Investment in *Shigella* surveillance in the Americas using a standardized methodology can contribute to accelerating *Shigella* vaccine development in consideration of regional preferences and optimal age of introduction.

## Author summary

*Shigella* infection is a leading cause of diarrhea worldwide and is annually responsible for over 81,000 deaths among children under five. Its burden and growing antibiotic resistance has led the World Health Organization to identify *Shigella* as a priority pathogen for vaccine development. There are many open discussions surrounding the development and rollout of a *Shigella* vaccine, including the pathogen serotypes that a vaccine should target and the age group by which complete immunization should be achieved. While the burden of *Shigella* cases and deaths has been shown to be highest in Africa and Asia, less is known about the burden of *Shigella* in the Americas. With this systematic review we aimed to synthesize the burden of *Shigella* diarrhea among children five years and younger in the Americas region to inform vaccine research, development, and regional policymakers. Our findings include gaps in what is currently known about the distribution of *Shigella* in the region and suggest approaches to improve data collection and reporting moving forward.

## Introduction

*Shigella* infection is a leading cause of diarrhea worldwide. In 2021, this Gram-negative bacterium was estimated to be responsible for over 81,000 deaths and over 7.3 million disability adjusted life years (DALYs) in children under five per year [1]. It is also estimated to contribute an additional 13,600 deaths annually due to malnutrition and stunted growth attributed to *Shigella’s* inflammatory and intestinal destruction mechanism of action [2]. Beyond its population health impacts, *Shigella* diarrhea is also associated with high household and healthcare system expenditure, which can exacerbate poverty and place significant demands on healthcare systems [3–6].

While the burden of *Shigella* cases and deaths has been shown to be highest in Africa and Asia, recent multi-site studies have also shown considerable burden in the Americas [7–11]. In 2018, the Global Pediatric Diarrhea Surveillance (GPDS) Network found that while rotavirus was the most common cause of diarrhea hospitalizations globally, *Shigella* was the leading cause in Central America, with an attributable fraction of 19.2% (95% CI: 11.4 – 28.1), and the third most common cause in South America with an attributable fraction of 11.8% (95% CI: 9.3 – 14.9), compared to 19.2% (95% CI: 12.7 – 28.8) in the Western Pacific and 15.4% (95% CI: 9.1 – 25.1) in South East Asia [12]. In 2012 the Etiology, Risk Factors and Interactions of Enteric Infections and Malnutrition and the Consequences for Child Health and Development (MAL-ED) study found a *Shigella* diarrhea incidence rate of 6.8 cases per 100 child years (95% CI: 3.4 – 11.5) in Brazil and 42.3 cases per 100 child years (95% CI: 32.5 – 53.0) in Peru [13]. Comparatively, between 2000 and 2004 a six country multi-center surveillance study in Asia found an incidence rate of 1.32 cases per 100 child years between 2000 and 2004 in children under five years of age [14]. In the United States, the burden of *Shigella* diarrhea is lower, with an estimated incidence of 18.15 cases per 100,000 children in the 1–4 year old age group [15]. Across the Americas, the burden of *Shigella* is particularly concerning given documented multi-drug resistant strains, including in the United States [16,17]. Data from 2014 to 2022 indicated that more than 80% of *Shigella sonnei* and *flexneri* isolates in the Americas were resistant to ampicillin, and there has been an increase in resistance to trimethoprim/sulfamethoxazole and increased non-susceptibility to ciprofloxacin from 0.2% to 5.7% within the same period [18,19].

With its increasing antibiotic resistance and continued high-burden, *Shigella* is a priority pathogen for vaccine development as evidenced by its moving up in priority ranking of the WHO Bacterial Priority Pathogens List, from 25^th^ in 2017 to 8^th^ in 2024 [20,21]. Several *Shigella* vaccines are in clinical development for the target population of young children with a Phase 3 trial underway and more anticipated in the coming years [22–26]. As policymakers in the Americas eventually consider whether a *Shigella* vaccine is appropriate for their setting, establishing an evidence-base on the burden estimates of *Shigella* diarrhea is critical [20]. This systematic review and meta-analysis synthesized the burden of *Shigella* diarrhea among children under 72 months (six years) in the Americas region. We gathered information on prevalence, incidence, and attributable fraction of *Shigella*-related diarrhea in this population, which is essential for informing vaccine research and development, regulation, and immunization-related policy making.

## Methods

We conducted a systematic review and meta-analyses to summarize the burden of *Shigella* diarrhea among children under 72 months of age in the Americas, excluding the U.S., Canada, and Greenland. Children in the low- and middle- income countries of the region were the focus due to the likelihood of a vaccine only being considered for pediatric routine vaccination in those contexts [27]. The protocol for this review was registered on Prospero (ID=CRD42024587471) and the PRISMA checklist for systematic reviews can be found in the supporting information (S1 Table) [28].

### Search strategy and selection criteria

We searched published and pre-print articles from PubMed, Embase, SciELO, CINAHL, Global Index Medicus, and Web of Science for this review. Articles written in English, Spanish, Portuguese, and French available in these databases from January 1, 2000 through July 23, 2024 were considered. Publications reporting only on data prior to 2000 were excluded to narrow the scope of this review to more recent burden estimates that can inform future policy. The search incorporated terms related to *Shigella* and diarrheal disease in children under 72 months of age (see S2 Table for complete search terms). We considered all interventional and observational study designs that enabled estimation of prevalence (proportion of diarrhea cases, hospitalizations, or deaths in which *Shigella* was identified), incidence (number of *Shigella*-specific diarrhea episodes per unit of person-time), and/or attributable fraction (the proportion of diarrhea episodes attributed to *Shigella* based on culture positivity, or molecular detection via PCR/qPCR) [29].

We restricted our inclusion to studies that confirmed *Shigella* infection by laboratory methods (culture, PCR, or qPCR) and that were conducted in countries of the Americas, excluding the U.S., Canada, and Greenland. Studies also had to report disaggregated data specifically for children aged under 72 months with symptomatic diarrheal disease to be included.

After removing duplicate articles, each title and abstract were independently screened for eligibility by two reviewers (MLS, AR, AM, PP) using Covidence systematic review software [30]. A third independent reviewer resolved any discrepancies between the two initial reviewers. Articles that could not be evaluated based on the abstract were automatically moved to full text review. Articles selected for full text review were screened utilizing the same dual review method described for titles and abstracts. At the full text review stage, the reason for exclusion of each ineligible article was recorded within Covidence. Reasons for exclusion were applied hierarchically beginning with incorrect geography, followed by data collection prior to 2000, study population without diarrhea, lack of laboratory confirmation of *Shigella*, absence of data disaggregated for children under 72 months, and insufficient information to calculate the required metrics.

Data were extracted from included articles utilizing a pre-specified data extraction template embedded in Covidence. Information on study design and methodology (e.g., follow-up duration and inclusion criteria), study location, number of overall children and/or stool samples and those with *Shigella* detected, key clinical descriptors (i.e., diarrhea definition), antibiotic use, and laboratory detection methods were extracted. Outcomes (prevalence, incidence, attributable fraction) and associated 95% confidence intervals (CIs) were extracted for the overall study population and any relevant subgroups reported by the manuscript authors such as disaggregated age groups, serotype, antibiotic resistance, healthcare setting, and study site urbanicity.

Publication quality was assessed using an adapted Joanna Briggs Institute (JBI) assessment tool (S3 Table) [31]. Quality assessment and data extraction occurred simultaneously using Covidence. All data extraction and quality assessment were conducted by a single reviewer and quality controlled by a second reviewer in Covidence.

### Data analysis

Descriptive tables were compiled to summarize the characteristics of all included studies. Forest plots of prevalence, attributable fraction, and incidence estimates were created to display data across studies according to key subgroups of interest, such as detection method and health care setting. Studies were categorized based on what was explicitly stated in the published article. If the authors did not specify a subgroup, for example urbanicity of the study site, the study would not be included in any category for that subgrouping. For studies that estimated prevalence using qPCR detection methods, we accepted their reported values regardless of the lower-limit of detection, quantitative threshold employed to assign detection, or the test manufacturer. In instances when only raw numbers were reported (such as number of *Shigella* cases and number of children presenting with diarrhea), *Shigella* prevalence estimates were calculated by the review team. For all prevalence estimates, the review team calculated 95% CIs using R, assuming a binomial distribution to standardize confidence intervals across studies. All incidence estimates were standardized by the review team to the rate of cases per 100 child-years. For all prevalence and attributable fraction outcome metrics, random effects meta-analyses were conducted for each subgroup of interest when relevant data from at least two studies in any subgroup were present using the “Meta” package in R [32]. A meta-analysis was not conducted across incidence estimates due to notable variability in estimation techniques throughout the literature, limiting comparability across estimates.

## Results

### Literature search results

**Fig 1:**
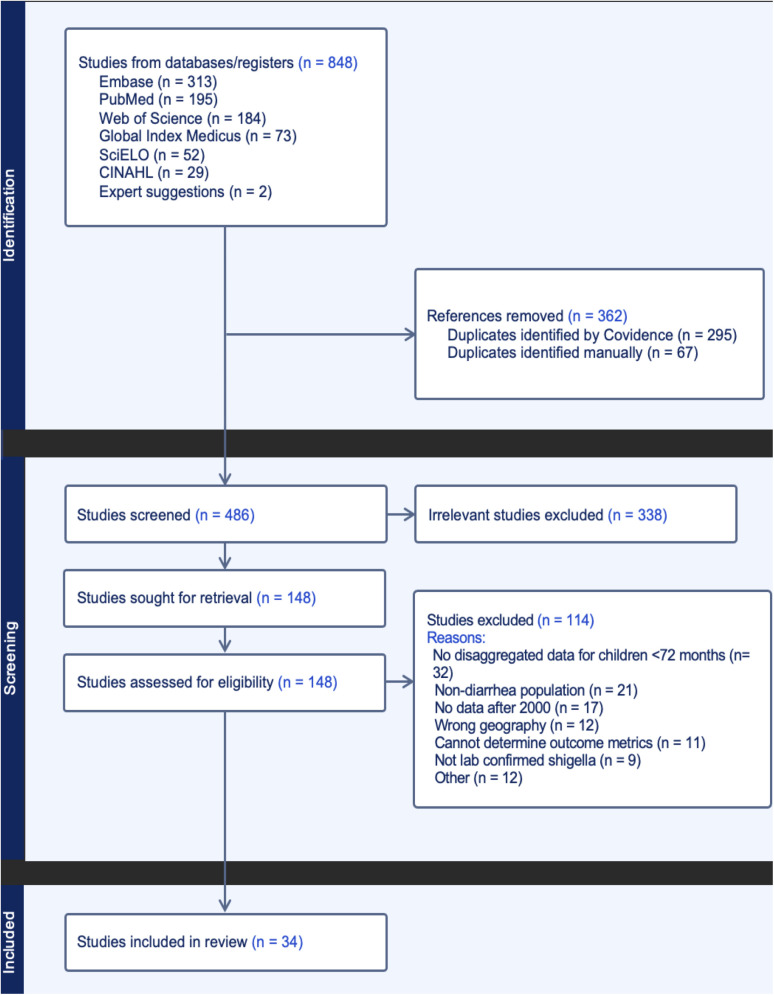
PRISMA diagram of literature search and screening results.

Our final search yielded 848 articles from all databases described previously. After removing duplicates, 486 articles remained for title and abstract screening. Of these, 338 titles/abstracts were excluded leaving 148 articles that met eligibility criteria for full-text review. During the full-text assessment, 114 studies were excluded resulting in a final 34 articles included in the review (Fig 1). Quality assessments of the 34 included studies can be found in S1 Fig.

**Fig 2:**
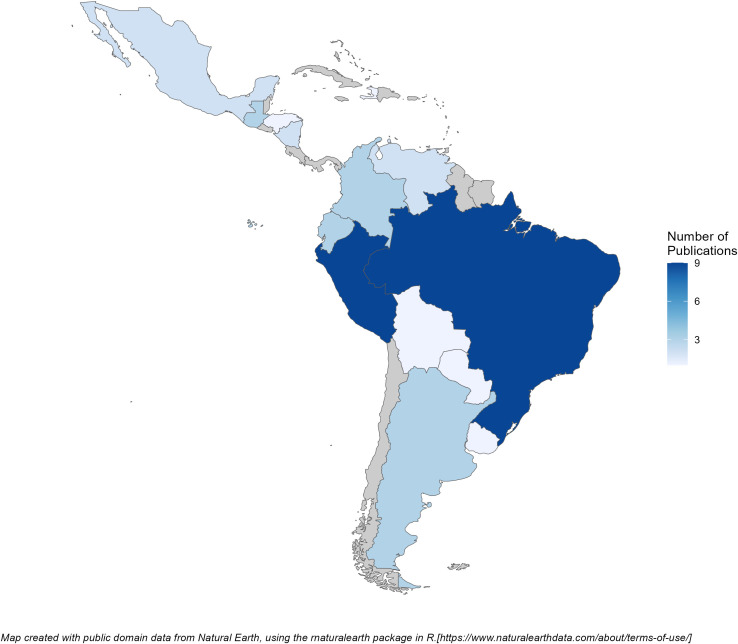
Heat map of included publications, by country in the Americas (excluding U.S., Canada, and Greenland). Made with public domain data from Natural Earth. Free vector and raster map data at https://www.naturalearthdata.com/. Terms of use available at https://www.naturalearthdata.com/about/terms-of-use/.

Key characteristics of the included studies are presented in Table 1, with a summary of study-level characteristics in Table 2. The data on the burden of *Shigella* infections originated from 14 countries, with Brazil and Peru contributing nine publications each (26.4%, respectively) (Fig 2). Among the included studies, 22 (64.7%) were cross-sectional studies. Nine studies (26%) used PCR/qPCR as a method of detection with the remaining using culture-based detection. The four studies that used qPCR specified varying cycle threshold (CT) value cutoffs/ lower limits of detection of less than 30 [53], less than 35 [12,13], and less than 38 [42] for *Shigella* detection, but did not report copies per milliliter. Studies used variable case definitions for diarrhea, with two explicitly excluding cases of bloody diarrhea [53,40]. Among those that included cases of bloody diarrhea, the reported proportion of such cases among all samples ranged considerably from 2.6% up to 30.8%.

**Table 1:** Key characteristics of all studies included in this review, listed alphabetically.

Author (Year)	Geography	Years of data collection	Health facility type	Diarrhea definition	Age range (months)	Lab confirmation	Sample size (% with visible blood)	Effect estimate type
Arvelo, et al. (2019) [33]	Guatemala	2008-09	Hospital, health center and health post	A case of acute diarrhea was defined as 3 or more loose stools in a 24hr period during the previous 7 days	<60	Culture	846 samples (not specified)	Prevalence
Balbachán, et al. (2007) [34]	Argentina	2004-05	Health center and post	Acute diarrhea for less than 72hrs	<60	Culture	590 samples (not specified)	Prevalence
Becker Dreps, et al. (2014) [35]	Nicaragua	2010-11	Community (Active surveillance)	Increase in stool frequency to at least 3 stools per 24hr period or as a substantial change in stool consistency (bloody, very loose, watery) after at least 3 diarrhea-free days	<60	Culture	337 samples (2.7% of all diarrhea samples)	Prevalence
Casabonne, et al. (2016) [36]	Argentina	2011-12	Hospital	Not specified	<72	PCR	766 samples (not specified)	Prevalence
Cermeño, et al. (2008) [37]	Venezuela	2004-05	Hospital and vaccination centers	Acute diarrhea: 3 or more loose or liquid stools within 24hrs or one bloody stool in a period of less than 14 days	<60	Culture	110 individuals (14.6% of all diarrhea samples)	Prevalence
Cohen, et al. (2022) [12]	Honduras, Peru, Ecuador, Bolivia, Nicaragua, Paraguay	2017-18^1^	Hospital based surveillance	Three or more loose stools in a 24hr period	<60	qPCR (<35 CT cut off)	1012 Individuals (not specified for population of interest)	Prevalence and Attributable fraction
Cornejo-Tapia, et al. (2017) [38]	Peru	2011-12	Hospital	Occurrence of diarrhea lasting less than 14 days along with symptoms such as vomiting, fever, dehydration and abdominal pain	<60	PCR	117 individuals (not specified)	Prevalence
Diniz-Santos, et al. (2005) [39]	Brazil	2002-03	Hospital	Not specified	<60	Culture and biochemical testing using EPM-MILI Citrate medium.	120 samples (not specified)	Prevalence
Gaensbauer, et al. (2019) [40]	Guatemala	2015-16	Hospital, health center and health post	Acute non-bloody diarrhea of < 72hrs duration and more than three liquid stools in the previous 24hrs	6-35	PCR	316 individuals (0% due to inclusion criteria)	Prevalence
Gambandé, et al. (2006) [41]	Argentina	2004-05	Hospital	Not specified	<60	Culture	182 samples (not specified)	Prevalence
Garcia Bardales, et al. (2022) [42]	Peru	2018-21	Tertiary and primary health facilities	Not specified	<24	qPCR (<38 CT cut off)	215 samples (not specified)	Prevalence
Hedge, et al. (2019) [43]	Guatemala	2007-12	Regional hospital, health centers and health posts	3 or more loose or liquid stools in a 24hr period with onset of illness within seven days before presenting to any participating facility	<60	Culture	151 *Shigella* cases (not specified for population of interest)	Incidence^2^
Kosek, et al. (2008) [44]	Peru	2002-06	Community (Active surveillance)	An overt change in a child as normal stool pattern characterized by an increase in the frequency to at least 3 unformed stools in a 24hr period	<72	Culture and biochemical testing.	3756 samples (30.8% of *Shigella* episodes)	Incidence and prevalence
Larrosa Haro, et al. (2010) [45]	Mexico	2006-07	Not specified	Not specified	<72	Culture	5459 samples (not specified)	Prevalence
Lima, et al. (2019) [46]	Brazil	2009-12	Community (Active surveillance)	Three or more liquid stools in the last 24hrs	2 - 36	PCR	596 Individuals (not specified)	Prevalence
Manrique-Abril, et al. (2006) [47]	Colombia	2004	Health center and health post	Three or more liquid or semi-liquid stools, or an atypical and bloody stool within 24hrs.	<60	Culture	129 samples (not specified)	Prevalence
Moreno, et al. (2010) [48]	Brazil	2000-01	Hospital	Three or more semisolid or liquid stools per day for 1–13 days	<24	Culture	290 individuals (13% of all diarrhea samples)	Prevalence
Moura, et al. (2012) [49]	Brazil	2010-11	Hospital	Presence of at least one stool with visible blood, or the three or more loose stools with an episode duration less than or equal to 14 days	<60	Culture	140 samples (not specified)	Prevalence
Nazate, et al. (2022) [50]	Ecuador	2021	Health center and health posts	Three or more mushy, semi-mushy or soft stools in 24hrs	<60	Culture	258 samples (not specified)	Prevalence
Ochoa et al. (2013) [51]	Peru	2008-11	Community (Active surveillance)	Presence of 3 or more watery stools in 24hrs or presence of blood in one or more stools.	12 - 18	Culture	555 individuals with 915 samples (2.6% of all diarrhea samples)	Prevalence
Ochoa T J, et al. (2009) [52]	Peru	2006-07	Community (Active surveillance)	Three liquid or semiliquid stools passed in a 24hr period or at least 1 loose stool with blood.	2 - 12	Culture	1034 individuals with 936 samples (not specified)	Prevalence
Operario, et al. (2017) [53]	Brazil	2013-14	Hospital	Non-bloody diarrhea of <14 days of duration.	<60	qPCR (<30 CT cut off)	557 samples (0% due to inclusion criteria)	Attributable fraction
Orlandi, et al. (2006) [54]	Brazil	2000-02	Hospital	Passage of three or more loose stools within the previous 24hrs that conformed to the shape of the container	<72	Culture and biochemical testing	470 samples (not specified)	Prevalence
Patzi-Vargas, et al. (2015) [55]	Mexico	2007-11	Hospital	Three or more liquid or semiliquid stools passed in a 24hr period	<60	Culture and biochemical testing	831 samples (11% of all diarrhea samples)	Prevalence
Peirano, et al. (2018) [56]	Uruguay	2012-15	Primary health facilities	Three or more discharges within 12hrs, or just one liquid or semiliquid stool including mucus, pus, or blood	<60	Culture	83 samples (6% of all diarrhea samples)	Prevalence
Perales, et al. (2002) [57]	Peru	2001	Health center and health post	Watery diarrhea (decreased consistency or increased frequency of bowel movements in 24hrs) with a duration of less than 72hrs	<24	Culture	248 samples (not specified)	Prevalence
Perez-Schael, et al. (2007) [58]	Venezuela	1998-2004	Hospital	Not specified	<60	Culture	69 deaths (not specified)	Prevalence
Platts-Mills, et al. (2018) [59]	Brazil and Peru	2009-12	Community (Active surveillance)	Maternal report of three or more loose stools in 24hrs, or one stool with visible blood	<24	qPCR (<35 CT cut off)	1672 samples (7.4% of *Shigella* episodes)	Incidence (calculated by study team)^3^
Platts-Mills, et al. (2015) [60]	Brazil, Peru	2009-14	Community (Active surveillance)	Maternal report of three or more loose stools in 24hrs, or one loose stool with visible blood	<24	Culture	2005 samples (5.5% of all diarrhea samples)	Attributable fraction
Sousa, et al. (2013) [61]	Brazil	2004-05	Hospital	Not specified beyond “acute diarrhea”	1 - 48	Culture and biochemical testing	157 samples (not specified)	Prevalence
Steenland, et al. (2013) [62]	Haiti	2012-13	Hospital	Three or more episodes of acute watery diarrhea within 24hrs, with onset of symptoms within the past 7 days	<60	Culture	319 individuals (not specified)	Prevalence
Urbina, et al. (2003) [63]	Colombia	1998-2000	Hospital and health center	Abnormal fecal discharge characterized by frequent‚ and at least three times per day‚ liquid or semi-liquid loose stools, accompanied by symptoms such as nausea, vomiting and fever and involving dehydration	<24	Culture	253 samples (59.5% of all diarrhea samples with bacteria isolates identified)	Prevalence
Uribe-Yepes, et al. (2009) [64]	Colombia	2006	Hospital and health centers	Acute diarrhea for less than 5 days	<60	Culture	180 samples (10.6% of all diarrhea samples)	Prevalence
Vieira, et al. (2007) [65]	Ecuador	2003-05	Community (Active surveillance)	Three or more loose stools in 24hrs	<60	PCR	133 individuals (not specified)	Prevalence

^1^Data updates from this study are publicly available on the World Health Organization website and were also extracted for this review.

^2^Calculated as number of new cases per 100,000 population of children less than 5. For each year, they calculated using mid-year population 2008–2012.

^3^Calculated as a product of number of episodes by attributable fraction divide by child years (expressed per 100 child years).

PCR: polymerase chain reaction test; qPCR: quantitative polymerase chain reaction test; CT: cycle threshold.

**Table 2:** Summary of key characteristics across included studies.

Publication characteristics	Number of articles	Percentage
***Country***		N = 34
Argentina	3	8.8
Brazil	9	26.4
Bolivia	1	2.9
Colombia	3	8.8
Ecuador	3	8.8
Guatemala	3	8.8
Haiti	1	2.9
Honduras	1	2.9
Mexico	2	5.8
Nicaragua	2	5.8
Paraguay	1	2.9
Peru	9	26.4
Uruguay	1	2.9
Venezuela	2	5.8
***Study type***		
Case control	5	14.7
Cohort	5	14.7
Cross sectional	22	64.7
RCT	2	5.8
***Primary Shigella detection method***		
Culture	25	73.6
PCR	5	14.7
qPCR	4	11.8
***Publication date***		
2000–2005	3	8.8
2006–2010	12	35.3
2011–2015	7	20.5
2016–2020	9	26.5
2020–2024^1^	3	8.8
***Reported metrics*** ^2^		
Attributable fraction	3	8.8
Incidence	3	8.8
Prevalence	30	88.2

^1^As of 18th July 2024.

^2^Two studies reported “prevalence and attributable fraction” and “prevalence and incidence” respectively.

### Prevalence results

**Fig 3:**
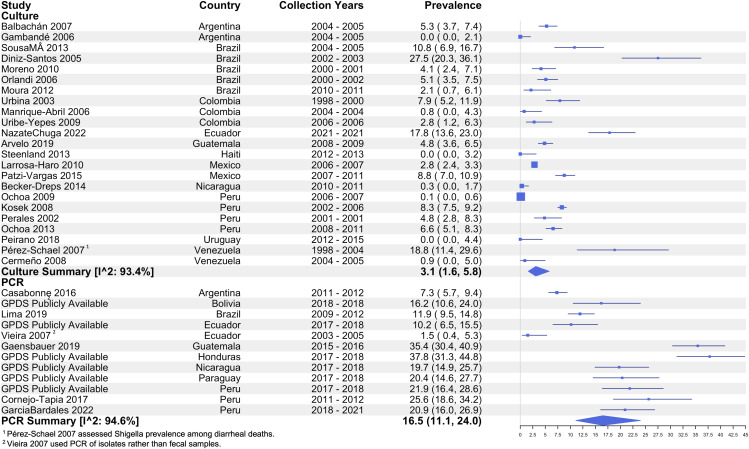
Prevalence of *Shigella* among diarrhea cases by detection method.

Across all studies that used culture as the method of detection and reported prevalence (n = 23), the pooled prevalence of *Shigella* among diarrhea cases was 3.1% (95% CI: 1.6 – 5.8, I^2^: 93.4%). The pooled prevalence among the 7 studies that used PCR/qPCR molecular methods for detection (using a variety of detection cut-offs) was more than five times higher at 16.5% (95% CI: 11.1 –24.0, I^2^: 94.6%). Among the culture-based studies, Diniz-Santos et al. (2005) [39] estimated the highest prevalence of 27.5% (95% CI: 20.3 – 36.1) among children <48 months in Brazil between 2002 and 2003 [39]. A few culture-based studies estimated a prevalence of zero from sites in Argentina, Haiti, and Uruguay in 2005, 2013, and 2015, respectively [56,62,66]. The highest prevalence estimate among PCR-based studies came from Honduras in 2017–2018 with 37.8% (95% CI: 31.3 – 44.8) among children 6–35 months while the lowest came from Ecuador in 2003–2005 at 1.5% (95% CI: 0.4 – 5.3) among children <60 months (Fig 3) [65].

**Fig 4:**
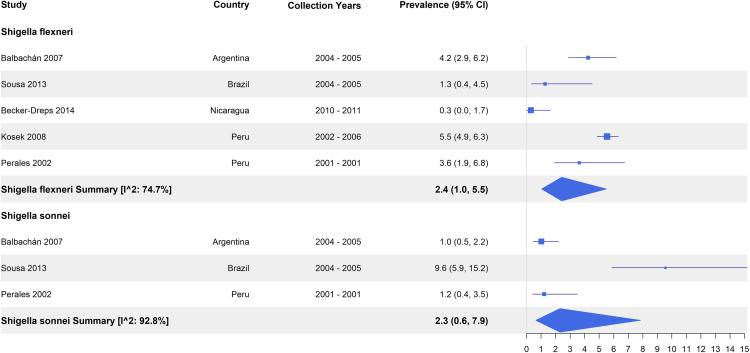
Prevalence of *Shigella* serotypes among diarrhea cases from culture-based studies.

A subset of culture-based studies reported the prevalence of *Shigella* specific to serotypes (n = 5). The pooled *Shigella flexneri* prevalence was 2.4% (95% CI: 1.0 – 5.5, I^2^: 74.7%) compared to 2.3% (95% CI: 0.6 – 7.9, I^2^: 92.8%) for *Shigella sonnei*. In Brazil, the prevalence of *Shigella flexneri* was lower than that of *Shigella sonnei*, while the opposite was true for Argentina and Peru [57,61,67] (Fig 4).

**Fig 5:**
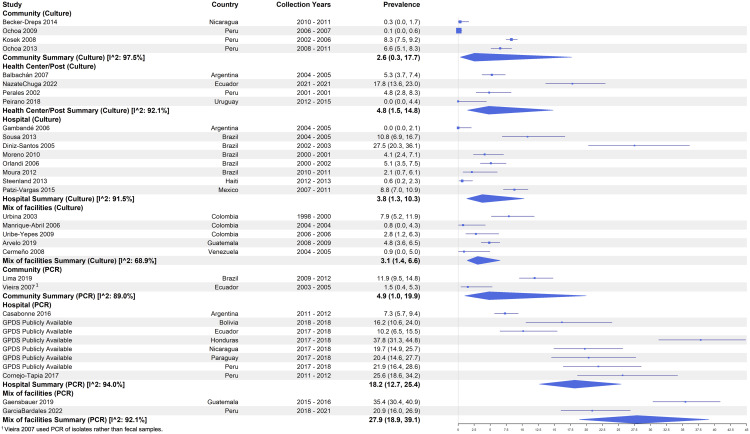
Prevalence of *Shigella* among diarrhea cases by detection method and health facility type.

**Fig 6:**
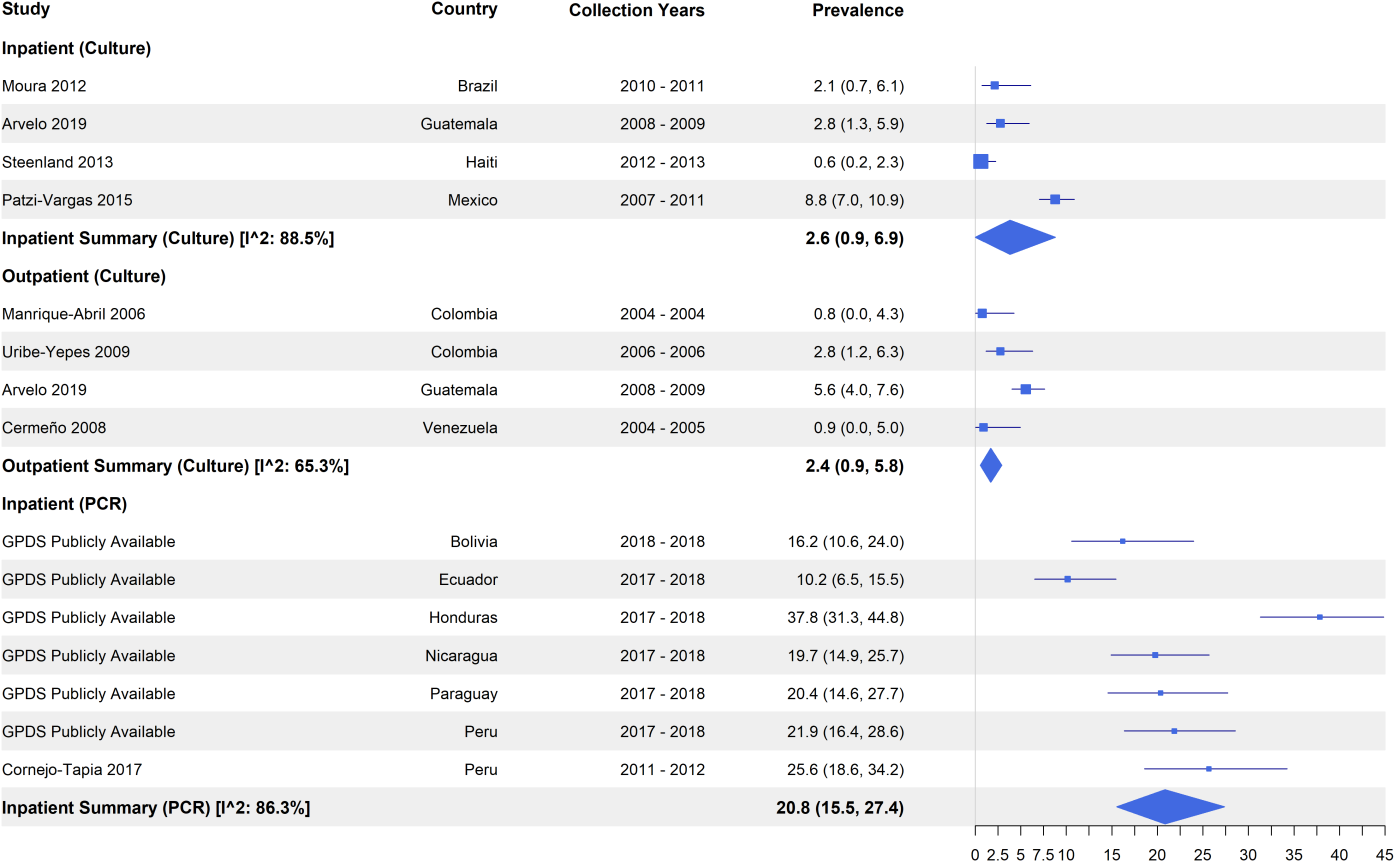
Prevalence of *Shigella* among diarrhea cases by detection method and hospital case type.

After disaggregating studies by healthcare facility type, the highest pooled prevalence estimates among culture-based studies came from four studies enrolling from health centers/posts (4.8%, 95% CI: 1.5 – 14.8, I^2^: 92.1%), followed by eight hospital-based studies at 3.8% (95% CI: 1.3 – 10.3, I^2^: 91.5%), five from a mix of facilities (3.1%, 95% CI: 1.4 – 6.6, I^2^: 68.9%) and four with active case finding in communities (2.6%, 95% CI: 0.3 –14.7, I^2^: 97.5%) (Fig 5). Among the culture-based hospital studies, a subset (n = 7) explicitly defined their population as either inpatient or outpatient cases and two reported for both inpatients and outpatients. Among the four studies with inpatient populations by culture, the pooled prevalence was 2.6% (95% CI: 0.9 – 6.9, I^2^: 88.5%), compared to 2.4% (95% CI: 0.9 – 5.8), I^2^: 65.3% among the five studies with outpatient hospital cases. PCR-based GPDS data for inpatients from six countries and one other study in Peru had an overall high prevalence of 20.8% (95% CI: 15.5 - 27.4, I^2^: 86.3%) (Fig 6).

**Fig 7:**
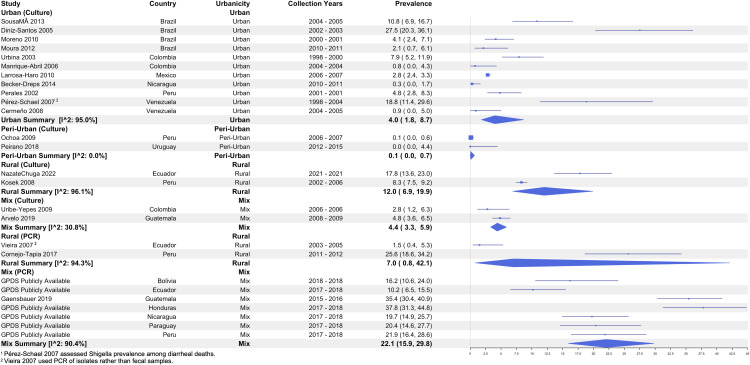
Prevalence of *Shigella* among diarrhea cases by detection method and urbanicity.

Sixteen culture and four PCR-based studies explicitly described the study setting as urban, peri-urban, or rural. While pooled prevalence estimates suggest that the burden of *Shigella* is generally lower in urban settings compared to rural settings, heterogeneity across studies resulted in large overlapping confidence intervals. Among culture-based studies, the pooled prevalence from urban studies was 4.0% (95% CI: 1.8 – 8.7, I^2^: 95.0%) while it was 12.0% (95% CI: 6.9 – 19.9, I^2^: 96.1%) among rural studies (Fig 7).

**Fig 8:**
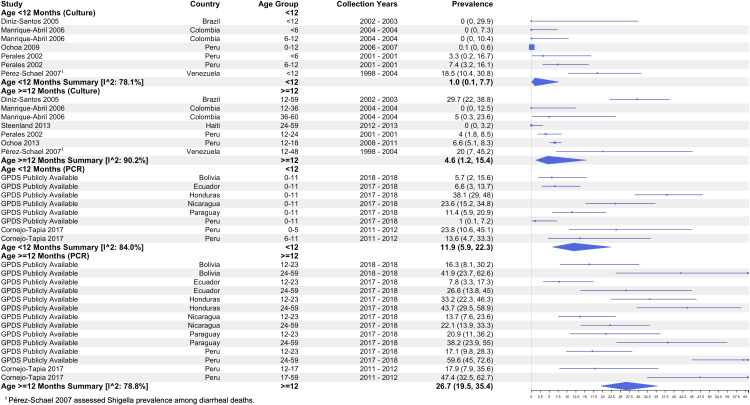
Prevalence of *Shigella* among diarrhea cases by detection method and age groups.

Studies used an array of age categories, most commonly by year (every 12 months). Other studies reported different intervals such as every two years, with three studies disaggregating ages less than 12 months old. Among culture-based results, the pooled prevalence estimate for children <12 months was 1.0% (95% CI: 0.1 – 7.7, I^2^: 78.1%) compared to 4.6% (95% CI: 1.2 – 15.4, I^2^: 90.2%) for children ≥12 months. Among PCR-based studies, the pooled prevalence estimate for children <12 months was 11.9% (95% CI: 5.9 – 22.3, I^2^: 84.0%) compared to 26.7% (95% CI: 19.5 – 35.4, I^2^: 78.8%) for children ≥12 months (Fig 8).

### Attributable fraction results

**Fig 9:**
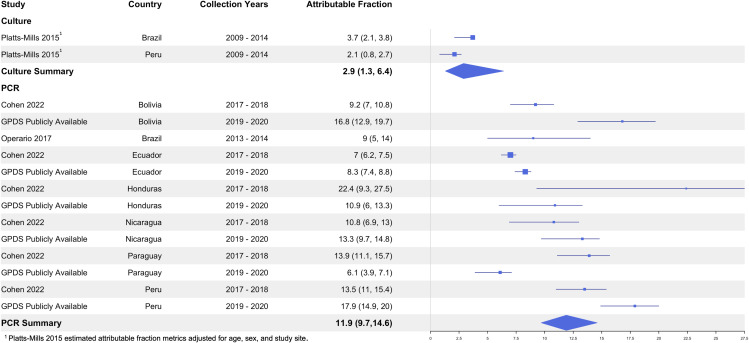
Attributable fraction of *Shigella* among diarrhea cases by detection method.

Only four studies (two of which from the same surveillance network) reported the burden of *Shigella* in terms of attributable fraction. Across the two culture-based estimates, the pooled attributable fraction was 2.9% (95% CI: 1.3 – 6.4, I^2^: 0.0%) [60]. Three PCR studies presented estimates across several countries, for which the pooled attributable fraction was 11.9% (95% CI: 9.7 – 14.6, I^2^: 48.8%) (Fig 9) [12].

### Incidence results

**Fig 10:**
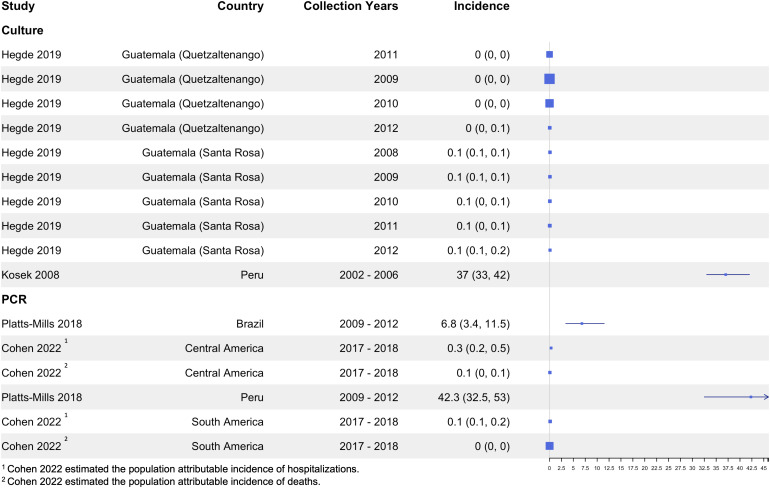
Incidence of *Shigella* among diarrhea cases by detection method.

Fewer studies reported incidence of *Shigella* diarrhea than prevalence and they used a variety of incidence method calculations ranging from direct ascertainment in a cohort study to facility-based studies that estimated incidence. Two studies conducted in Iquitos, Peru showed particularly high incidence of 37.0 per 100 child-years (95% CI: 33.0 – 42.0) from Kosek (2008) [44], which was a culture-based study, and 42.3 per 100 child-years (95% CI: 32.5 – 53.0) from Platts-Mills (2018) [60], a PCR-based study (Fig 10) conducted in the same study area.

## Discussion

This review aimed to describe the burden of *Shigella* among children five years and younger with diarrhea in the Americas to inform surveillance efforts, vaccine research and development investments, and ultimately aid policymakers with vaccine adoption decision-making. The Americas will be an important region to target for eventual *Shigella* vaccines due to its high burden and history of rapid vaccine adoption. Across the 34 studies reviewed, we found notable high prevalence, attributable fraction, and incidence estimates of *Shigella* diarrhea highlighting the burden of this Gram-negative bacteria among children living in the Americas.

This systematic review disaggregated all identified study estimates by detection method, given well known discrepancies between culture and PCR/qPCR-based sensitivities. Among culture-based studies, our meta-analysis identified a pooled *Shigella* diarrhea prevalence of 2.4%, which is slightly lower than estimates from systematic reviews in other regions. Namely, a systematic review of the prevalence of *Shigella* in Africa, which did not disaggregate by detection method, found a pooled prevalence of 5.9% [11]. Another systematic review in Southeast Asia, again without detection-disaggregated estimates, identified a pooled prevalence of 5% among children <5 years [68]. Within the Americas region, there are clear differences across burden estimates, however no clear patterns by country emerged that could indicate geographic vaccination priorities.

PCR/qPCR methods consistently result in burden estimates several times higher those from culture in the literature [69]. Our meta-analysis of *Shigella* prevalence from studies that used qPCR or PCR showed estimates more than five times higher than those by culture, with a pooled prevalence of 16.5%. Although a concern with PCR/qPCR methods is the detection of inactive DNA, culture-negative/molecularly identified *Shigella* cases appear to have similar severity of diarrhea and similar improvements with antibiotic treatment to culture-confirmed *Shigella* suggesting similar clinical relevance [70,71]*.* De-tuning quantitative PCR/qPCR methods, such as by choosing a higher-quantity infection threshold for *Shigella* detection based on cut-offs that distinguish diarrhea case from asymptomatic control status, may successfully distinguish clinically relevant *Shigella* cases from asymptomatic carriage at the individual case level. At a population level, attributable fraction calculations have been used to account for asymptomatic carriage estimates in controls. Only one molecular detection-based study (of nine) in this review used a de-tuned detection threshold [53], therefore the pooled PCR/qPCR molecular estimates in this review may be overstating the true *Shigella*-attributed diarrhea burden . Alignment on the threshold for the number of copies of the *ipaH* gene warranting likely attribution of diarrhea to *Shigella* across research studies would be an important step to minimize methodological heterogeneity between studies using molecular methods.

A small subset of studies reported prevalence of *Shigella* serotypes, specifically the two leading serotypes, *S. flexneri* and *S. sonnei*. It is hypothesized that serotype distributions shift from *S. flexneri* to *S. sonnei* predominance with economic development as well as water and sanitation infrastructure improvements. This shift is hypothesized to be due to the reduced presence *of Plesiomonas shigelloides* in contaminated water*,* a Gram-negative bacterium that shares some antigens with *S. sonnei* conferring immunity against *S. sonnei* [15,51,52,54,55]. Our meta-analyses of culture-based estimates show that the overall burden of both serotypes across the region are comparable. At the country level, in 2013 this trend held true for Brazil while Argentina and Peru had higher prevalence of *S. flexneri* in 2007 and 2002, respectively. The overall lack of *Shigella* serotype data in published literature makes it difficult to discern these trends over time and contribute to discussions about serotype targeting for vaccine development.

We further disaggregated prevalence study estimates into other subgroups of interest, while maintaining separation by detection method, including the type of health facility from which the study population was identified, and whether the study facilities were in urban or rural settings. Our meta-analysis found higher prevalence of *Shigella* in health center and hospital settings compared to community-based active case finding studies (see S2 Fig for the health facility subgroup forest plots with attributable fraction and incidence estimates). That *Shigella* diarrhea is more severe than other causes and that care-seeking tends to identify more severe diarrhea is consistent with other studies [1]. We found a lower pooled prevalence of *Shigella* among studies conducted in urban settings compared to rural settings, which aligns with findings from other studies that have similarly suggested higher prevalence of *Shigella* in rural settings, such as in a surveillance-based study of *Shigella* over 20 years in Bangladesh [72,73]. Uncertainty in pooled estimates with few rural prevalence estimates or differences in infrastructure between settings may explain the heterogeneity.

Age of optimal *Shigella* vaccine introduction remains an open discussion with general agreement that full protection would ideally occur prior to 12 months of age [74–76]. However, an increasingly crowded infant vaccine schedule may prove challenging. To explore variations in burden, we disaggregated pooled burden estimates by age and found that the prevalence of *Shigella* was higher among children above 12 months of age compared to younger children, consistent with other studies [77]. However, the majority of the data is cross-sectional and therefore does not explain how much *Shigella* diarrhea could be averted by a vaccine introduced at different ages. The MAL-ED study [60] ascertained age of first infection across seven countries, including Peru, and found the median time of first infection with *Shigella* to be 14 months of age [77]. Only two culture-based studies disaggregated the prevalence of *Shigella* among children <6 months compared to those 6–12 months old. While Manrique-Abril et al. [47] found comparable prevalence across these two groups, Perales et al. [57] demonstrated a burden more than twice as high among 6–12 month-olds compared to children <6 months old, a finding consistent with age-stratified *Shigella* prevalence in MAL-ED [47,57,77]. These data suggest that studies reporting *Shigella* burden in 0–12-month-old children may mask a notable burden among the 6–12-month sub-group. The one included study that presented age-stratified *Shigella* diarrhea incidence rates (Kosek 2008)[44] reported a peak *Shigella* diarrhea incidence of 50 cases/100 child-years in 12–23 month-olds but also noted a non-negligible burden in 6–11 months-olds (22 cases/100 child-years) [44]. Given uncertainty about precise timing of *Shigella* vaccine introduction, future studies should disaggregate their reporting of *Shigella* burden data in 3–6-month age categories, particularly among children under two years of age.

Incidence rates were less commonly reported in studies, likely due to the additional complexity of enumerating a population at risk and ensuring all possible cases are accounted for [29]. In the two studies that utilized a standard prospective cohort study design with active diarrhea episode ascertainment, including that which did not lead to care-seeking, incidence rates of *Shigella* diarrhea were exceptionally high compared to other incidence estimates, irrespective of detection method [44,59]. Incidence rates provide a better understanding of disease burden as they are unbiased by health care seeking and social exclusion, which affect ascertainment in health care centers, particularly in highly marginalized populations [78].

Incidence studies that focused on health-facility ascertainment of cases, both outpatient and inpatient, and exclusively inpatients, had lower incidence rates, consistent with rarer, but costly, more severe disease [12,43]. Health care system costs, and costs averted by introduction of a *Shigella* vaccine by preventing outpatient and inpatient visits, will be key factors for country decision makers to consider when faced with a potentially effective and available *Shigella* vaccine (see S2 Fig for forest plot of attributable fraction estimates among only inpatient populations). The cost of an eventual *Shigella* vaccine will also be an important factor for policy-makers. Larger vaccine markets help to drive down prices through initiatives like pooled procurement, making vaccines more accessible for the lowest income settings. This review highlights that *Shigella* diarrhea appears to be distributed across the Americas and expanding surveillance in the region may further demonstrate a universal and notable *Shigella* burden throughout the region.

Beyond cost-effectiveness, vaccine developers must also consider that country immunization programs are becoming saturated, which is reflected in the number of vaccination visits and shots per visit, where the same number of health workers are asked to manage increasingly complex vaccination schedules [74]. As such, combination vaccine strategies based on identified and validated regional priority vaccine targets have been raised as the way forward for *Shigella* vaccines [74,79]. This review may further inform regional decision makers on the relative burden of *Shigella* and therefore its relevance to the research and development of a combination vaccine.

The majority of studies included in this review were conducted from populations within Brazil and Peru, with minimal representation in the rest of the Americas region resulting in limited generalizability of results. In addition to data from more countries, sub-national data is needed to inform a better understanding of *Shigella* epidemiology in the Americas. The high burden in Iquitos, Peru, confirmed by multi-country studies including sites in Asia and Africa, has led some to believe the Amazon region is a particularly high-risk place for *Shigella* compared to the rest of the Americas due to infrastructure- and climate-related factors that may promote bacteria growth [44,59]. Few studies estimated the burden of *Shigella* in other communities within the Amazon region, limiting the ability to generalize the burden observed in Iquitos to other areas of the Amazon in Peru, as well as Brazil, Colombia, and Ecuador (see S4 Table for all burden estimates from study sites in the Amazon region). It is also noteworthy that incidence rates from sites selected for research studies may not reflect the true population-based incidence in the region because areas with known high disease burden are more likely to be selected for study and intervention. Conversely, children in the lowest resourced and most isolated settings who generally have the lowest access to state-of-the-art diagnostics may not be represented in the research studies reported in this review.

Beyond limited variation in the geographic scope of studies in the region, another limitation of our systematic review is the overall heterogeneity of reporting practices across articles, resulting in the exclusion of valid and useful data across various subgroup analyses, for example, prevalence by age. In some cases, the lack of data available for comparison across studies resulted in reduced power of the meta-analyses to estimate pooled metrics and thus produced imprecise corresponding confidence intervals. Some studies excluded children with previous antibiotic use and/or children with co-infections which further limited comparability across studies*.* Regarding seasonality of *Shigella*, this review did not restrict study inclusion by duration of recruitment, which could result in comparison of specific seasonal burden estimates to those from year-round data collection. Finally, most of the studies included in this review (n = 27) were facility-based thus limiting understanding of the burden of *Shigella* to the region’s health systems, rather than that of the general population. Despite the overall limited number of studies that met inclusion criteria and the varying data collection and reporting practices of those included studies, we were able to produce several informative meta-analyses to identify pooled burden estimates across key subgroups relevant to vaccine development and eventual deployment considerations.

Overall, our findings on the burden of *Shigella* in the Americas emphasize the need to further study *Shigella* incidence and prevalence*.* Importantly, data would be most useful with a standardized detection methodology, such as a uniform qPCR platform with a de-tuned threshold, across a variety of geographic settings, including throughout the Amazon region, and to report data disaggregated by subpopulations of interest to contribute to key vaccine discussions. These findings may incentivize investment in *Shigella* surveillance and contribute to accelerating *Shigella* vaccine development, and eventual uptake, in consideration of regional preferences, including multi-pathogen vaccine development strategies.

## Supporting information

S1 Fig Quality assessment of 34 included studies. (DOCX)

S2 Fig Additional forest plots of Shigella burden. (DOCX)

S1 Table PRISMA 2020 checklist for systematic reviews. (DOCX)

S2 Table Database search strings. (DOCX)

S3 Table Adapted Joanna Briggs Institute (JBI) quality assessment tool. (DOCX)

S4 Table Burden estimates from study sites in the Amazon region. (DOCX)
