# Supplementary figures and images for: Burden of Shigella among children with diarrhea in the Americas: A systematic review and meta-analysis
Source: PLoS Negl Trop Dis. 2025 Aug 18;19(8):e0013393. doi: 10.1371/journal.pntd.0013393 (PMC12413091; doi:10.1371/journal.pntd.0013393)

**S1 Fig: Quality Assessment of Included Studies**


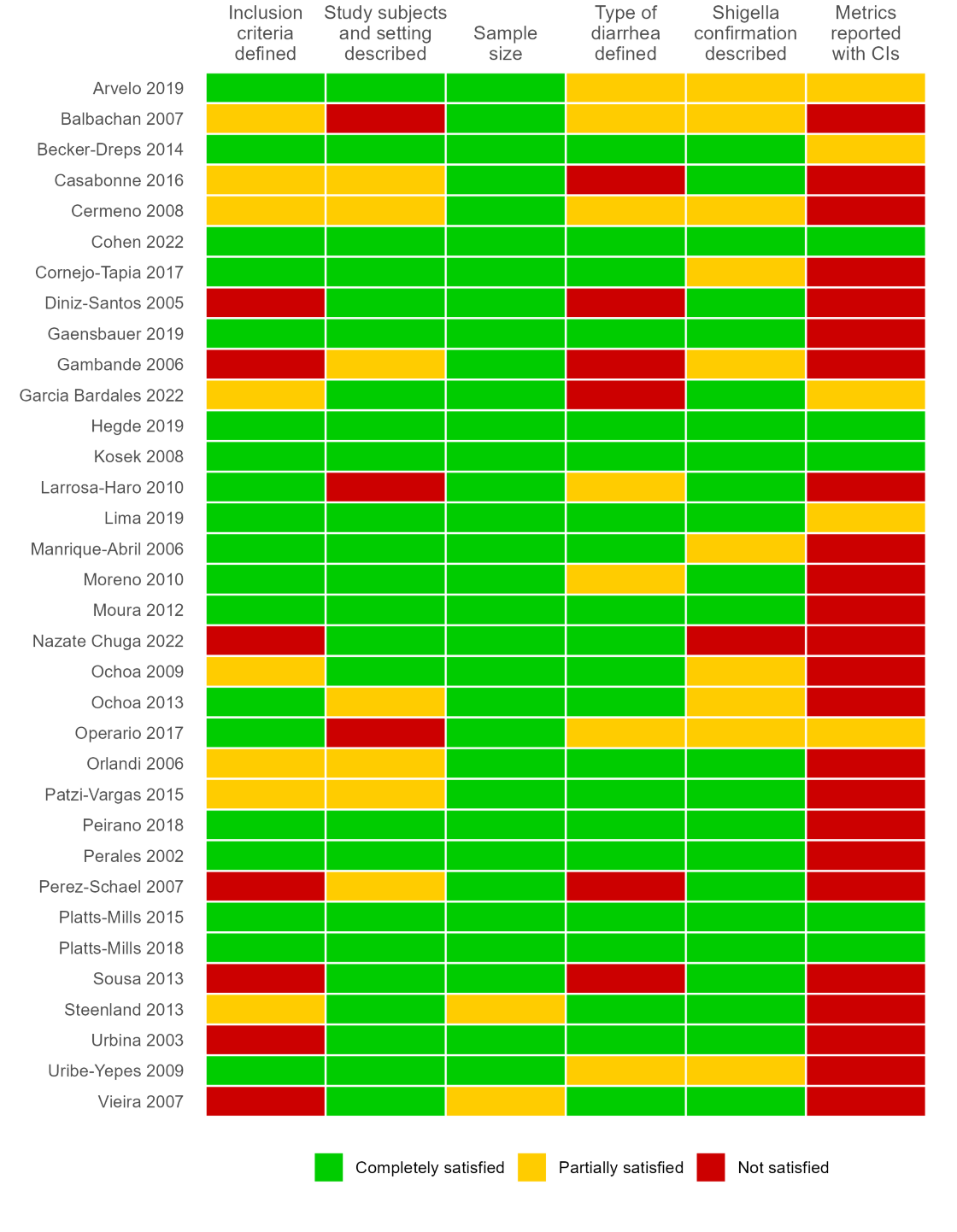

Supplement: S1 Fig — (DOCX) [file pntd.0013393.s001.docx]
