# Supplementary material for: Burden of Shigella among children with diarrhea in the Americas: A systematic review and meta-analysis
Source: PLoS Negl Trop Dis. 2025 Aug 18;19(8):e0013393. doi: 10.1371/journal.pntd.0013393 (PMC12413091; doi:10.1371/journal.pntd.0013393)
Supplement: S2 Fig — (DOCX) [file pntd.0013393.s002.docx]

**S2 Fig:  Additional Forest Plots of *Shigella* Burden**

*Attributable fraction of Shigella among diarrhea cases by diagnostic method and health facility type.*


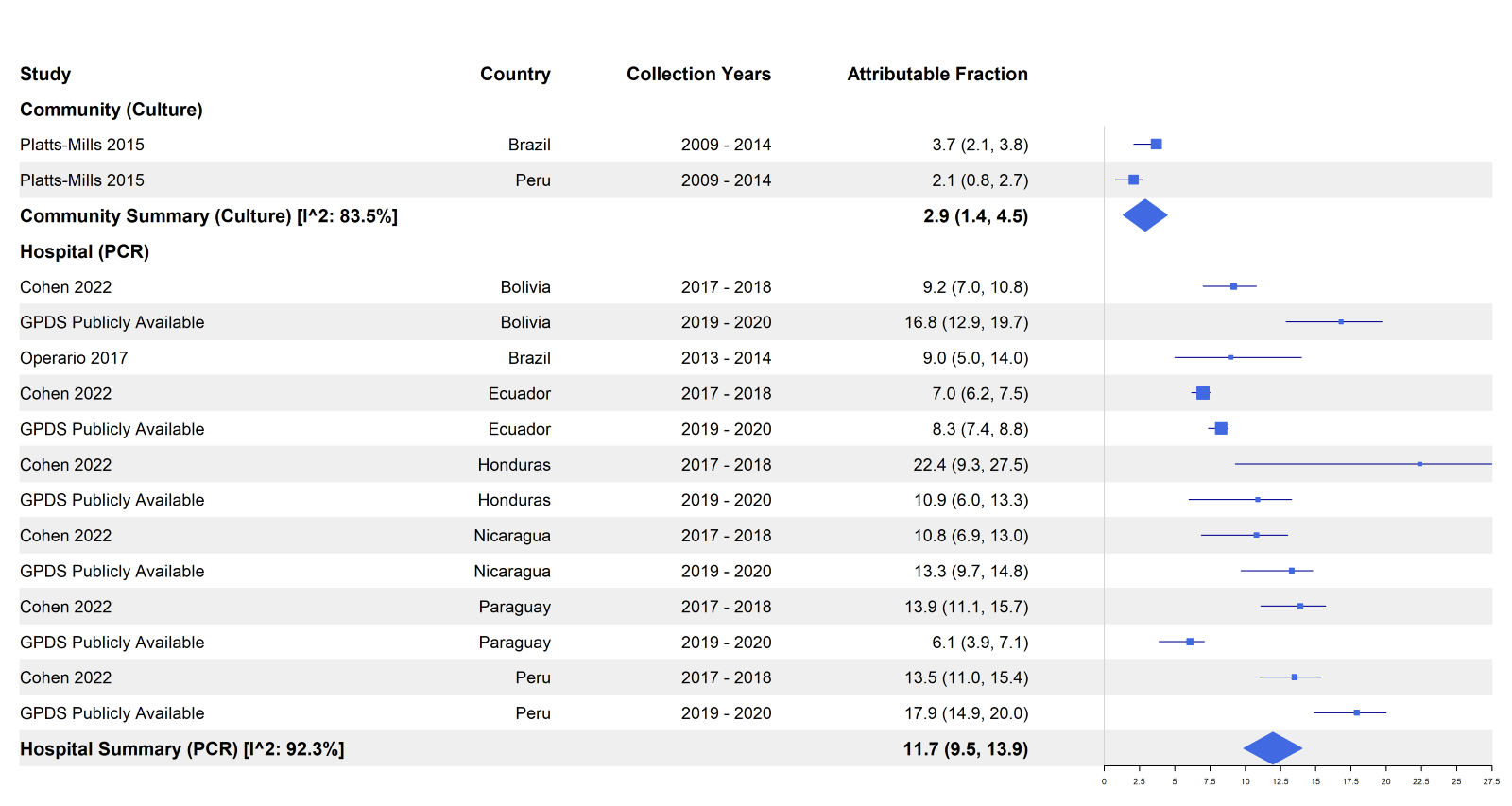


*Incidence of Shigella among diarrhea cases by diagnostic method and health facility type.*


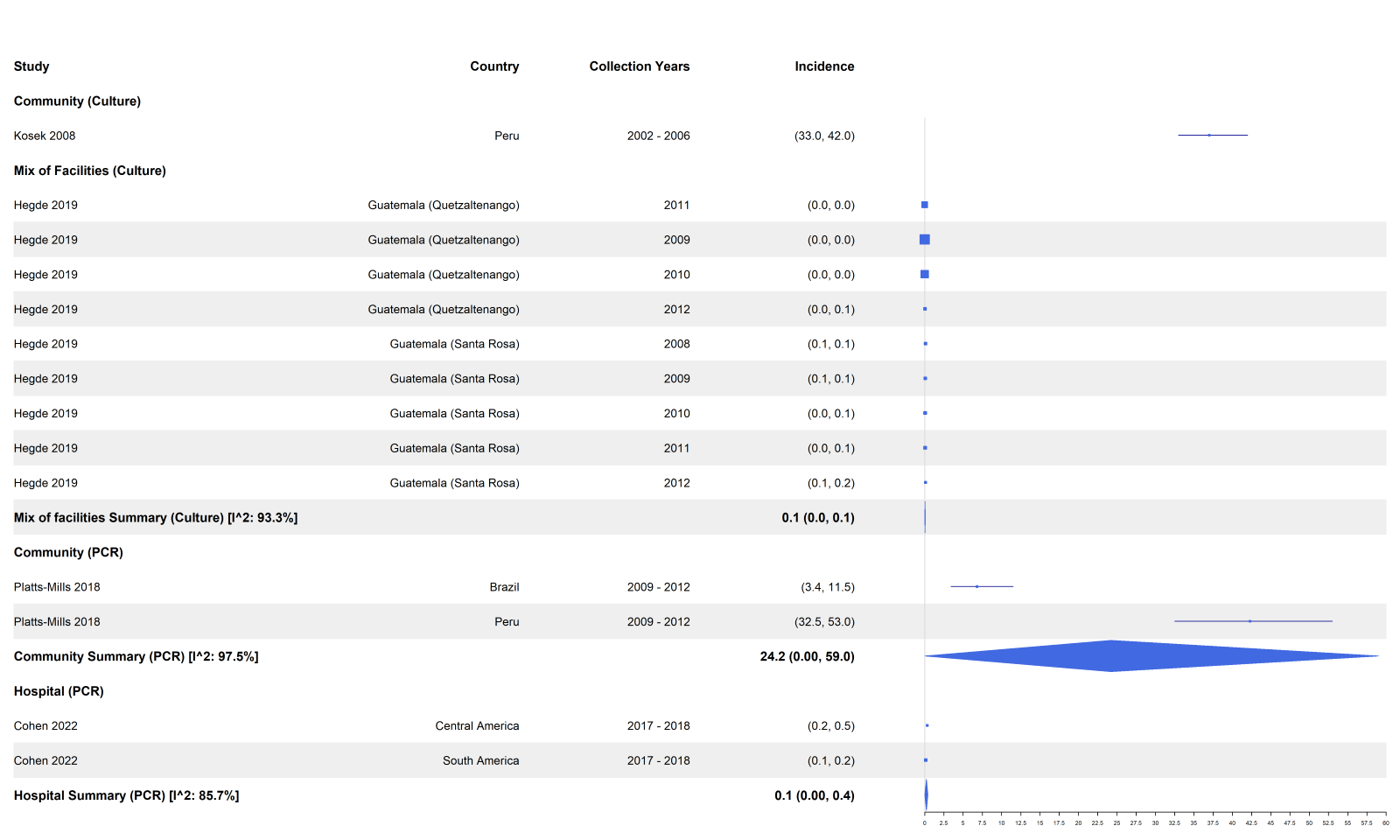


*Attributable fraction of Shigella among diarrhea cases by diagnostic method and hospital case type.*


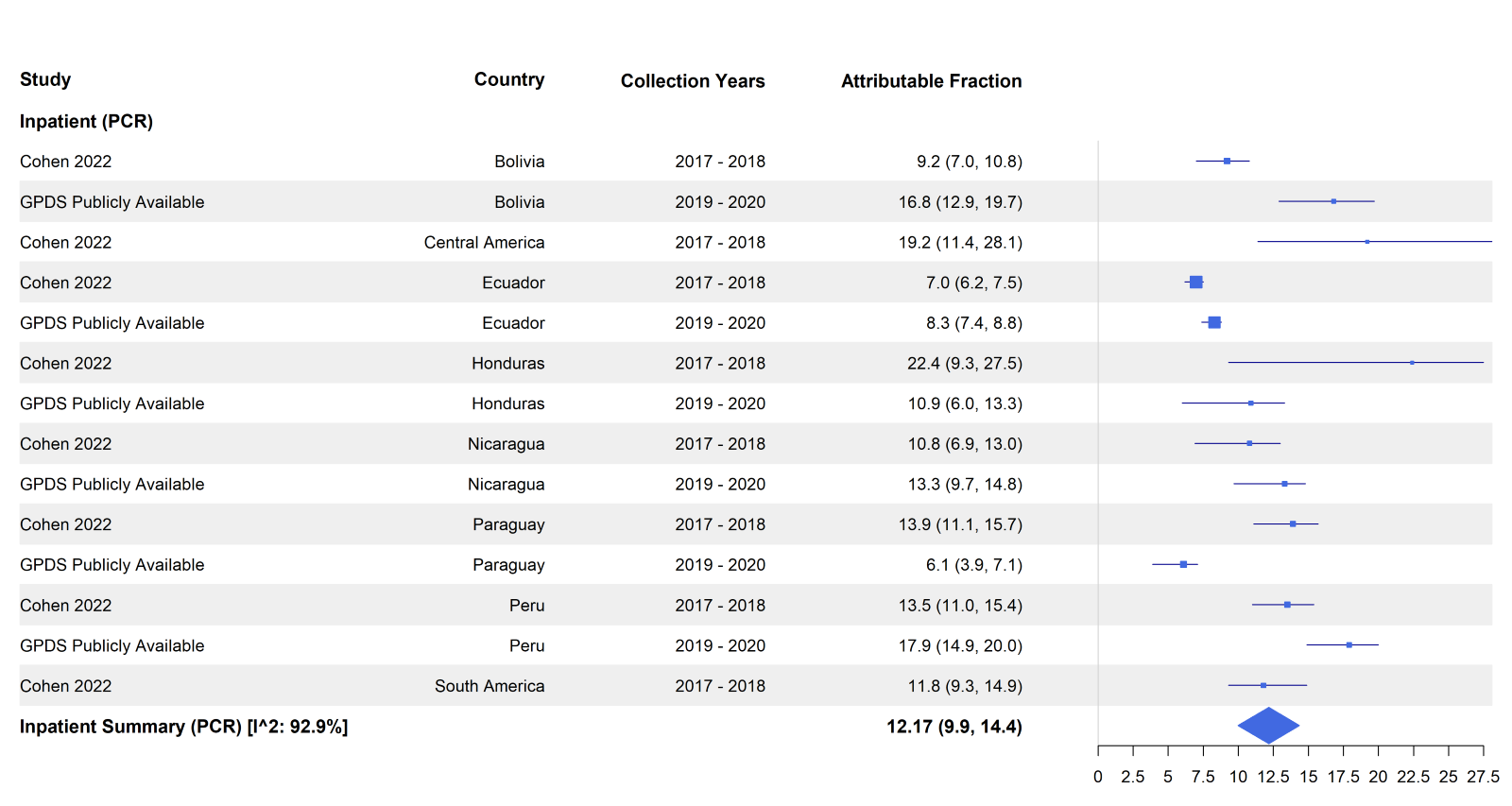


**References**

1. Platts-Mills JA, Liu J, Rogawski ET, Kabir F, Lertsethtakarn P, Siguas M, et al. Use of quantitative molecular diagnostic methods to assess the aetiology, burden, and clinical characteristics of diarrhoea in children in low-resource settings: a reanalysis of the MAL-ED cohort study. The Lancet Global Health. 2018;6: e1309–e1318. doi:10.1016/S2214-109X(18)30349-8
2. Cohen AL, Platts-Mills JA, Nakamura T, Operario DJ, Antoni S, Mwenda JM, et al. Aetiology and incidence of diarrhoea requiring hospitalisation in children under 5 years of age in 28 low-income and middle-income countries: findings from the Global Pediatric Diarrhea Surveillance network. BMJ Glob Health. 2022;7: e009548. doi:10.1136/bmjgh-2022-009548
3. Operario DJ, Platts-Mills JA, Nadan S, Page N, Seheri M, Mphahlele J, et al. Etiology of Severe Acute Watery Diarrhea in Children in the Global Rotavirus Surveillance Network Using Quantitative Polymerase Chain Reaction. The Journal of Infectious Diseases. 2017;216: 220–227. doi:10.1093/infdis/jix294
4. Kosek M, Yori PP, Pan WK, Olortegui MP, Gilman RH, Perez J, et al. Epidemiology of Highly Endemic Multiply Antibiotic-Resistant Shigellosis in Children in the Peruvian Amazon. Pediatrics. 2008;122: e541–e549. doi:10.1542/peds.2008-0458
5. Hegde S, Benoit SR, Arvelo W, Lindblade K, López B, McCracken JP, et al. Burden of laboratory-confirmed shigellosis infections in Guatemala 2007-2012: results from a population-based surveillance system. BMC Public Health. 2019;19: 474. doi:10.1186/s12889-019-6780-7
6. Platts-Mills JA, Babji S, Bodhidatta L, Gratz J, Haque R, Havt A, et al. Pathogen-specific burdens of community diarrhoea in developing countries: a multisite birth cohort study (MAL-ED). The Lancet Global Health. 2015;3: e564–e575. doi:10.1016/S2214-109X(15)00151-5
