# Supplementary material for: Burden of Shigella among children with diarrhea in the Americas: A systematic review and meta-analysis
Source: PLoS Negl Trop Dis. 2025 Aug 18;19(8):e0013393. doi: 10.1371/journal.pntd.0013393 (PMC12413091; doi:10.1371/journal.pntd.0013393)
Supplement: S2 Table — (DOCX) [file pntd.0013393.s004.docx]

**S2 Table: Database Search Strings**

PubMed; NCBI  (1946-)

|  | Concept: Shigella | Concept: incidence/prevalence | Concept:  PAHO countries |
| --- | --- | --- | --- |
| Subject Headings  (MeSH) | "Shigella"[Mesh] "Dysentery, Bacillary"[Mesh] | Incidence[Mesh:noexp]Prevalence[Mesh:noexp]  Cross-Sectional Studies[Mesh:noexp] "Case-Control Studies"[Mesh] "Disease Hotspot"[Mesh] epidemiology[sh] | Latin America[Mesh] Central America[Mesh] South America[Mesh] Mexico[Mesh] Caribbean Region[Mesh] Atlantic Islands[Mesh] Caribbean People[Mesh]  Central American People[Mesh] South American People[Mesh] Netherlands Antilles[Mesh] |
| Free text terms  (searched in title, abstract, and author keywords [tiab] or in [text words](https://pubmed.ncbi.nlm.nih.gov/help/#tw) [tw])) | Searched in title, abstract, and author keywords [tiab]  shigella*[tiab] shigellosis[tiab]  "bacillary dysentery"[tiab]  "shiga bacillus dysentery"[tiab] | Searched in title, abstract, and author keywords [tiab]  incidence[tiab] incidences[tiab] prevalence[tiab]  prevalences[tiab] cross-sectional[tiab]  "association study"[tiab:~2] "association studies"[tiab:~2] "ecological studies"[tiab]  "ecological study"[tiab] transversal[tiab]  ((case[tiab] OR cases[tiab]) AND (control[tiab] OR controls[tiab])) case-comparison[tiab]  case-compeer[tiab] case-base[tiab]  case-referent[tiab] "attributable fraction*"[tiab]  "attributable risk*"[tiab]    Searched in title only  association[ti] associations[ti] | Searched in [text words](https://pubmed.ncbi.nlm.nih.gov/help/#tw) [tw] **"Latin America*"[tw] "Central America*"[tw] "South America*"[tw]** Anguilla*[tw] Antigua*[tw] Barbados[tw] Barbadian*[tw] Barbuda*[tw] Argentin*[tw] Aruba*[tw] Baham*[tw] Belize*[tw] Bolivia*[tw] Brazil*[tw] Brasil*[tw] "Virgin Island*"[tw] "Cayman Island*"[tw] Chile*[tw] Colombia*[tw] "Costa Rica*"[tw] Cuba*[tw] Curaçao*[tw] Dominica*[tw] Ecuador*[tw] "El Salvador"[tw] Salvadoran*[tw] "Falkland Island*"[tw] Falklander*[tw] Grenada*[tw] Guadeloup*[tw] Guatemala[tw] Guatemalan*[tw] Guian*[tw] Guyan*[tw] Haiti*[tw] Haïti*[tw] Ayiti*[tw] Hispaniola[tw] Hondura*[tw] Jamaica*[tw] Martinique[tw] Martiniquais*[tw] Martinican*[tw] Mexic*[tw] Méxic*[tw] Montserrat*[tw] "Netherlands Antille*"[tw] Nicaragua*[tw] Panama*[tw] Panamá*[tw] Paraguay*[tw] Paraguái*[tw] Peru*[tw] Perú*[tw] "Puerto Rico*"[tw] "Puerto Rican*"[tw] "Saint Kitts"[tw] "St Kitts"[tw] Nevis[tw] Kittitian*[tw] Nevisian*[tw] "Saint Lucia*"[tw] "St Lucia*"[tw] "Saint Vincent"[tw] "St Vincent"[tw] Vincentian*[tw] Grenadines[tw] "Sint Maarten*"[tw] "Saint Martin*"[tw] "St Martin*"[tw] "Saint Bart*"[tw] "St Bart*"[tw] Barthélemois[tw] "Saint Pierre"[tw] "St Pierre"[tw] Saint-Pierrais[tw] Pierrian*[tw] Miquelon*[tw] Surinam*[tw] Trinidad*[tw] Tobago*[tw] "Turks and Caicos"[tw] Uruguay*[tw] Venezuela*[tw] Caribbean[tw]  "West Indies"[tw] |

("Shigella"[Mesh] OR "Dysentery, Bacillary"[Mesh] OR shigella*[tiab] OR shigellosis[tiab] OR "bacillary dysentery"[tiab] OR "shiga bacillus dysentery"[tiab])

 AND

(Incidence[Mesh:noexp] OR Prevalence[Mesh:noexp] OR Cross-Sectional Studies[Mesh:noexp] OR "Case-Control Studies"[Mesh] OR "Disease Hotspot"[Mesh] OR epidemiology[sh] OR incidence[tiab] OR incidences[tiab] OR prevalence[tiab] OR prevalences[tiab] OR cross-sectional[tiab] OR "association study"[tiab:~2] OR "association studies"[tiab:~2] OR "ecological studies"[tiab] OR "ecological study"[tiab] OR transversal[tiab] OR association[ti] OR associations[ti] OR ((case[tiab] OR cases[tiab]) AND (control[tiab] OR controls[tiab])) OR case-comparison[tiab] OR case-compeer[tiab] OR case-base[tiab] OR case-referent[tiab] OR "attributable fraction*"[tiab] OR "attributable risk*"[tiab])

 AND

(Latin America[Mesh] OR Central America[Mesh] OR South America[Mesh] OR Mexico[Mesh] OR Caribbean Region[Mesh] OR Atlantic Islands[Mesh] OR Caribbean People[Mesh] OR Central American People[Mesh] OR South American People[Mesh] OR Netherlands Antilles[Mesh] OR **"Latin America*"[tw] OR "Central America*"[tw] OR "South America*"[tw] OR** Anguilla*[tw] OR Antigua*[tw] OR Barbados[tw] OR Barbadian*[tw] OR Barbuda*[tw] OR Argentin*[tw] OR Aruba*[tw] OR Baham*[tw] OR Belize*[tw] OR Bolivia*[tw] OR Brazil*[tw] OR Brasil*[tw] OR "Virgin Island*"[tw] OR "Cayman Island*"[tw] OR Chile*[tw] OR Colombia*[tw] OR "Costa Rica*"[tw] OR Cuba*[tw] OR Curaçao*[tw] OR Dominica*[tw] OR Ecuador*[tw] OR "El Salvador"[tw] OR Salvadoran*[tw] OR "Falkland Island*"[tw] OR Falklander*[tw] OR Grenada*[tw] OR Guadeloup*[tw] OR Guatemala[tw] OR Guatemalan*[tw] OR Guian*[tw] OR Guyan*[tw] OR Haiti*[tw] OR Haïti*[tw] OR Ayiti*[tw] OR Hispaniola[tw] OR Hondura*[tw] OR Jamaica*[tw] OR Martinique[tw] OR Martiniquais*[tw] OR Martinican*[tw] OR Mexic*[tw] OR Méxic*[tw] OR Montserrat*[tw] OR "Netherlands Antille*"[tw] OR Nicaragua*[tw] OR Panama*[tw] OR Panamá*[tw] OR Paraguay*[tw] OR Paraguái*[tw] OR Peru*[tw] OR Perú*[tw] OR "Puerto Rico*"[tw] OR "Puerto Rican*"[tw] OR "Saint Kitts"[tw] OR "St Kitts"[tw] OR Nevis[tw] OR Kittitian*[tw] OR Nevisian*[tw] OR "Saint Lucia*"[tw] OR "St Lucia*"[tw] OR "Saint Vincent"[tw] OR "St Vincent"[tw] OR Vincentian*[tw] OR Grenadines[tw] OR "Sint Maarten*"[tw] OR "Saint Martin*"[tw] OR "St Martin*"[tw] OR "Saint Bart*"[tw] OR "St Bart*"[tw] OR Barthélemois[tw] OR "Saint Pierre"[tw] OR "St Pierre"[tw] OR Saint-Pierrais[tw] OR Pierrian*[tw] OR Miquelon*[tw] OR Surinam*[tw] OR Trinidad*[tw] OR Tobago*[tw] OR "Turks and Caicos"[tw] OR Uruguay*[tw] OR Venezuela*[tw] OR Caribbean[tw] OR "West Indies"[tw])

AND (2000:2024[pdat])

Limits: years limited to 2000-present

**Results**: 188 results as of July 18, 2024

*Missing geographies added 7 results as of Aug. 26, 2024*

**Embase; Elsevier (1947-)**

|  | Concept: Shigella | Concept: incidence/prevalence | Concept:  PAHO countries |
| --- | --- | --- | --- |
| Subject Headings  (Emtree) | 'shigella'/exp  'shigellosis'/exp | 'incidence'/exp ‘'disease hotspot'/exp 'infection rate'/exp  'prevalence'/exp 'cross-sectional study'/exp **'epidemiology'/lnk** | 'South and Central America'/exp 'Mexico'/exp 'Caribbean (person)'/exp'Central American'/exp ‘'South American'/exp 'Central American'/exp |
| Free text terms | Searched in title, abstract, and author keywords (:ti,ab,kw)  shigella* shigellosis "bacillary dysentery" "shiga bacillus dysentery" | Searched in title, abstract, and author keywords (:ti,ab,kw)  Incidence incidences prevalence  Prevalences cross-sectional  **(association NEAR/3 (study OR studies))** "ecological studies"  "ecological study" transversal  ((case OR cases) AND (control OR controls)) case-comparison  case-compeer case-base  case-referent "attributable fraction*" "attributable risk*"    Searched in title only  Association associations | Searched in title, abstract, author keywords, subject terms, and journal title (:ti,ab,kw,de,jt)  **"Latin America*" "Central America*" "South America*"** Anguilla*  Antigua* Barbados Barbadian* Barbuda* Argentin* Aruba* Baham*  Belize* Bolivia* Brazil* Brasil* "Virgin Island*" "Cayman Island*" Chile* Colombia* "Costa Rica*" Cuba* Curaçao* Dominica* Ecuador* "El Salvador" Salvadoran* 'Falkland Island*' Falklander*  Grenada* Guadeloup* Guatemala Guatemalan* Guian* Guyan*  Haiti* Haïti* Ayiti* Hispaniola Hondura* Jamaica* Martinique Martiniquais* Martinican* Mexic* Méxic* Montserrat* "Netherlands Antille*" Nicaragua* Panama* Panamá* Paraguay* Paraguái* Peru* Perú* "Puerto Rico*" "Puerto Rican*" "Saint Kitts" "St Kitts" Nevis Kittitian* Nevisian* "Saint Lucia*" "St Lucia*" "Saint Vincent" "St Vincent" Vincentian* Grenadines "Sint Maarten*" 'Saint Martin*' 'St Martin*' 'Saint Bart*' 'St Bart*' Barthélemois 'Saint Pierre' 'St Pierre' Saint-Pierrais Pierrian* Miquelon* Surinam* Trinidad* Tobago* "Turks and Caicos" Uruguay* Venezuela* Caribbean "West Indies" |

('shigella'/exp OR 'shigellosis'/exp OR (shigella* OR shigellosis OR "bacillary dysentery" OR "basillary dysentery" OR "bacterial dysentery" OR "shiga bacillus dysentery"):ti,ab,kw)

 AND

('incidence'/exp OR 'disease hotspot'/exp OR 'infection rate'/exp OR 'prevalence'/exp OR 'cross-sectional study'/exp **OR 'epidemiology'/lnk OR** (incidence OR incidences OR prevalence OR prevalences OR **cross-sectional OR (association NEAR/3 (study OR studies)) OR "ecological study" OR "ecological studies" OR transversal OR** ((case OR cases) AND (control OR controls)) OR case-comparison OR case-compeer OR case-base OR case-referent OR "attributable fraction*" OR "attributable risk*"**):ti,ab,kw OR (association OR associations):ti)**

 AND

('South and Central America'/exp OR 'Mexico'/exp OR 'Caribbean (person)'/exp OR 'Central American'/exp OR 'South American'/exp OR 'Central American'/exp OR (**"Latin America*" OR "Central America*" OR "South America*"** OR Anguilla* **OR** Antigua* OR Barbados OR Barbadian* OR Barbuda* OR Argentin* OR Aruba* OR Baham* OR Belize* OR Bolivia* OR Brazil* OR Brasil* OR "Virgin Island*" OR "Cayman Island*" OR Chile* OR Colombia* OR "Costa Rica*" OR Cuba* OR Curaçao* OR Dominica* OR Ecuador* OR "El Salvador" OR Salvadoran* OR 'Falkland Island*' OR Falklander* OR Grenada* OR Guadeloup* OR Guatemala OR Guatemalan* OR Guian* OR Guyan* OR Haiti* OR Haïti* OR Ayiti* OR Hispaniola OR Hondura* OR Jamaica* OR Martinique OR Martiniquais* OR Martinican* OR Mexic* OR Méxic* OR Montserrat* OR "Netherlands Antille*" OR Nicaragua* OR Panama* OR Panamá* OR Paraguay* OR Paraguái* OR Peru* OR Perú* OR "Puerto Rico*" OR "Puerto Rican*" OR "Saint Kitts" OR "St Kitts" OR Nevis OR Kittitian* OR Nevisian* OR "Saint Lucia*" OR "St Lucia*" OR "Saint Vincent" OR "St Vincent" OR Vincentian* OR Grenadines OR "Sint Maarten*" OR 'Saint Martin*' OR 'St Martin*' OR 'Saint Bart*' OR 'St Bart*' OR Barthélemois OR 'Saint Pierre' OR 'St Pierre' OR Saint-Pierrais OR Pierrian* OR Miquelon* OR Surinam* OR Trinidad* OR Tobago* OR "Turks and Caicos" OR Uruguay* OR Venezuela* OR Caribbean OR "West Indies"):ti,ab,kw,de,jt)

AND [2000-2024]/py

NOT ([conference abstract]/lim OR [conference paper]/lim OR [conference review]/lim)

Limits: years limited to 2000-present; no conference materials

**Results**: 260 results as of July 18, 2024

*Missing geographies added 4 results as of Aug. 26, 2024*

**SciELO (Clarivate)**

|  | Concept: Shigella | Concept: incidence/prevalence |
| --- | --- | --- |
| Topic  (searches title, abstract, author keywords, Keywords Plus®) | Searched in Topic  shigella* shigellosis "bacillary dysentery" "shiga bacillus dysentery" | Searched in Topic  Incidence incidences prevalence prevalences cross-sectional **(association NEAR/2 (study OR studies))** "ecological studies" "ecological study" Transversal ((case OR cases) AND (control OR controls)) case-comparison case-compeer case-base case-referent "attributable fraction*" "attributable risk*"    Searched in title only  Association associations |

Running note: Run string from the Advanced Search. Set date limits on Advanced Search page or in the results sidebar.

TS=(shigella* OR shigellosis OR "bacillary dysentery" OR "shiga bacillus dysentery")

 AND

( TS=(incidence OR incidences OR prevalence OR prevalences OR cross-sectional OR **(association NEAR/2 (study OR studies)) OR** "ecological studies" OR "ecological study" OR transversal OR ((case OR cases) AND (control OR controls)) OR case-comparison OR case-compeer OR case-base OR case-referent OR "attributable fraction*" OR "attributable risk*") OR TI=(association OR associations) )

Limits: years limited to 2000-present

**Results**: 52 results as of July 18, 2024

**CINAHL Complete (Cumulative Index to Nursing and Allied Health Literature); EBSCO (1937-)**

|  | Concept: Shigella | Concept: incidence/prevalence | Concept:  PAHO countries |
| --- | --- | --- | --- |
| Subject Headings (MH) | "Shigella"  "Dysentery, Bacillary" | "Incidence" "Prevalence" "Cross Sectional Studies" "Case Control Studies+" "Disease Hotspot" MW "EP" | "Latin America" "Central America+" "South America+" "Mexico" "West Indies+" "Atlantic Islands+" "Caribbean Persons+" "Central Americans" "South Americans+" "Mexicans+" |
| Geographic subset |  |  | (ZZ "mexico & central/south america") |
| Free text terms  (searched in Title & Abstract; keyword not a searchable field) | Searched in title and abstract (TI, AB)  shigella*  shigellosis  "bacillary dysentery"  "shiga bacillus dysentery" | Searched in title and abstract (TI, AB)  Incidence incidences prevalence prevalences cross-sectional **(association N2 (study OR studies))** "ecological studies" "ecological study" Transversal ((case OR cases) AND (control OR controls)) case-comparison case-compeer  case-base case-referent "attributable fraction*" "attributable risk*"    Searched in title only (TI)  association  associations | Searched in title, abstract, author keywords, subject terms, and journal title (TI, AB, DE,SO)  **"Latin America*" "Central America*" "South America*"** Anguilla* Antigua* Barbados Barbadian* Barbuda* Argentin* Aruba* Baham* Belize* Bolivia* Brazil* Brasil* "Virgin Island*" "Cayman Island*" Chile* Colombia* "Costa Rica*" Cuba* Curaçao* Dominica* Ecuador* "El Salvador" Salvadoran* "Falkland Island*" Falklander* Grenada* Guadeloup* Guatemala Guatemalan* Guian* Guyan* Haiti* Haïti* Ayiti* Hispaniola Hondura* Jamaica* Martinique Martiniquais* Martinican* Mexic* Méxic* Montserrat* "Netherlands Antille*" Nicaragua* Panama* Panamá* Paraguay* Paraguái* Peru* Perú* "Puerto Rico*" "Puerto Rican*" "Saint Kitts" "St Kitts" Nevis Kittitian* Nevisian* "Saint Lucia*" "St Lucia*" "Saint Vincent" "St Vincent" Vincentian* Grenadines "Sint Maarten*" "Saint Martin*" "St Martin*" "Saint Bart*" "St Bart*" Barthélemois "Saint Pierre" "St Pierre" Saint-Pierrais Pierrian* Miquelon* Surinam* Trinidad* Tobago* "Turks and Caicos" Uruguay* Venezuela* Caribbean "West Indies" |

Running note: On Advanced Search screen, make sure the following boxes are not checked: "Apply related words", "Also search within the full text of the articles", "Apply equivalent subjects"

To limit by publication type, select all relevant types in the Source Types options in the results sidebar.

(MH("Shigella" OR "Dysentery, Bacillary") OR TI(shigella* OR shigellosis OR "bacillary dysentery" OR "shiga bacillus dysentery") OR AB(shigella* OR shigellosis OR "bacillary dysentery" OR "shiga bacillus dysentery"))

 AND

(MH("Incidence" OR "Prevalence" OR "Cross Sectional Studies" OR "Case Control Studies+" OR "Disease Hotspot") OR MW "EP" OR TI(incidence OR incidences OR prevalence OR prevalences OR cross-sectional OR **(association N2 (study OR studies)) OR** "ecological studies" OR "ecological study" OR transversal OR ((case OR cases) AND (control OR controls)) OR case-comparison OR case-compeer OR case-base OR case-referent OR "attributable fraction*" OR "attributable risk*") OR AB(incidence OR incidences OR prevalence OR prevalences OR cross-sectional OR **(association N2 (study OR studies)) OR** "ecological studies" OR "ecological study" OR transversal OR ((case OR cases) AND (control OR controls)) OR case-comparison OR case-compeer OR case-base OR case-referent OR "attributable fraction*" OR "attributable risk*") OR TI(association OR associations))

AND

(MH("Latin America" OR "Central America+" OR "South America+" OR "Mexico" OR "West Indies+" OR "Atlantic Islands+" OR "Caribbean Persons+" OR "Central Americans" OR "South Americans+" OR "Mexicans+") OR (ZZ "mexico & central/south america") OR **TI("Latin America*" OR "Central America*" OR "South America*" OR** Anguilla* OR Antigua* OR Barbados OR Barbadian* OR Barbuda* OR Argentin* OR Aruba* OR Baham* OR Belize* OR Bolivia* OR Brazil* OR Brasil* OR "Virgin Island*" OR "Cayman Island*" OR Chile* OR Colombia* OR "Costa Rica*" OR Cuba* OR Curaçao* OR Dominica* OR Ecuador* OR "El Salvador" OR Salvadoran* OR "Falkland Island*" OR Falklander* OR Grenada* OR Guadeloup* OR Guatemala OR Guatemalan* OR Guian* OR Guyan* OR Haiti* OR Haïti* OR Ayiti* OR Hispaniola OR Hondura* OR Jamaica* OR Martinique OR Martiniquais* OR Martinican* OR Mexic* OR Méxic* OR Montserrat* OR "Netherlands Antille*" OR Nicaragua* OR Panama* OR Panamá* OR Paraguay* OR Paraguái* OR Peru* OR Perú* OR "Puerto Rico*" OR "Puerto Rican*" OR "Saint Kitts" OR "St Kitts" OR Nevis OR Kittitian* OR Nevisian* OR "Saint Lucia*" OR "St Lucia*" OR "Saint Vincent" OR "St Vincent" OR Vincentian* OR Grenadines OR "Sint Maarten*" OR "Saint Martin*" OR "St Martin*" OR "Saint Bart*" OR "St Bart*" OR Barthélemois OR "Saint Pierre" OR "St Pierre" OR Saint-Pierrais OR Pierrian* OR Miquelon* OR Surinam* OR Trinidad* OR Tobago* OR "Turks and Caicos" OR Uruguay* OR Venezuela* OR Caribbean OR "West Indies") OR AB(**"Latin America*" OR "Central America*" OR "South America*" OR** Anguilla* OR Antigua* OR Barbados OR Barbadian* OR Barbuda* OR Argentin* OR Aruba* OR Baham* OR Belize* OR Bolivia* OR Brazil* OR Brasil* OR "Virgin Island*" OR "Cayman Island*" OR Chile* OR Colombia* OR "Costa Rica*" OR Cuba* OR Curaçao* OR Dominica* OR Ecuador* OR "El Salvador" OR Salvadoran* OR "Falkland Island*" OR Falklander* OR Grenada* OR Guadeloup* OR Guatemala OR Guatemalan* OR Guian* OR Guyan* OR Haiti* OR Haïti* OR Ayiti* OR Hispaniola OR Hondura* OR Jamaica* OR Martinique OR Martiniquais* OR Martinican* OR Mexic* OR Méxic* OR Montserrat* OR "Netherlands Antille*" OR Nicaragua* OR Panama* OR Panamá* OR Paraguay* OR Paraguái* OR Peru* OR Perú* OR "Puerto Rico*" OR "Puerto Rican*" OR "Saint Kitts" OR "St Kitts" OR Nevis OR Kittitian* OR Nevisian* OR "Saint Lucia*" OR "St Lucia*" OR "Saint Vincent" OR "St Vincent" OR Vincentian* OR Grenadines OR "Sint Maarten*" OR "Saint Martin*" OR "St Martin*" OR "Saint Bart*" OR "St Bart*" OR Barthélemois OR "Saint Pierre" OR "St Pierre" OR Saint-Pierrais OR Pierrian* OR Miquelon* OR Surinam* OR Trinidad* OR Tobago* OR "Turks and Caicos" OR Uruguay* OR Venezuela* OR Caribbean OR "West Indies") OR DE(**"Latin America*" OR "Central America*" OR "South America*" OR** Anguilla* OR Antigua* OR Barbados OR Barbadian* OR Barbuda* OR Argentin* OR Aruba* OR Baham* OR Belize* OR Bolivia* OR Brazil* OR Brasil* OR "Virgin Island*" OR "Cayman Island*" OR Chile* OR Colombia* OR "Costa Rica*" OR Cuba* OR Curaçao* OR Dominica* OR Ecuador* OR "El Salvador" OR Salvadoran* OR "Falkland Island*" OR Falklander* OR Grenada* OR Guadeloup* OR Guatemala OR Guatemalan* OR Guian* OR Guyan* OR Haiti* OR Haïti* OR Ayiti* OR Hispaniola OR Hondura* OR Jamaica* OR Martinique OR Martiniquais* OR Martinican* OR Mexic* OR Méxic* OR Montserrat* OR "Netherlands Antille*" OR Nicaragua* OR Panama* OR Panamá* OR Paraguay* OR Paraguái* OR Peru* OR Perú* OR "Puerto Rico*" OR "Puerto Rican*" OR "Saint Kitts" OR "St Kitts" OR Nevis OR Kittitian* OR Nevisian* OR "Saint Lucia*" OR "St Lucia*" OR "Saint Vincent" OR "St Vincent" OR Vincentian* OR Grenadines OR "Sint Maarten*" OR "Saint Martin*" OR "St Martin*" OR "Saint Bart*" OR "St Bart*" OR Barthélemois OR "Saint Pierre" OR "St Pierre" OR Saint-Pierrais OR Pierrian* OR Miquelon* OR Surinam* OR Trinidad* OR Tobago* OR "Turks and Caicos" OR Uruguay* OR Venezuela* OR Caribbean OR "West Indies") OR SO(**"Latin America*" OR "Central America*" OR "South America*" OR** Anguilla* OR Antigua* OR Barbados OR Barbadian* OR Barbuda* OR Argentin* OR Aruba* OR Baham* OR Belize* OR Bolivia* OR Brazil* OR Brasil* OR "Virgin Island*" OR "Cayman Island*" OR Chile* OR Colombia* OR "Costa Rica*" OR Cuba* OR Curaçao* OR Dominica* OR Ecuador* OR "El Salvador" OR Salvadoran* OR "Falkland Island*" OR Falklander* OR Grenada* OR Guadeloup* OR Guatemala OR Guatemalan* OR Guian* OR Guyan* OR Haiti* OR Haïti* OR Ayiti* OR Hispaniola OR Hondura* OR Jamaica* OR Martinique OR Martiniquais* OR Martinican* OR Mexic* OR Méxic* OR Montserrat* OR "Netherlands Antille*" OR Nicaragua* OR Panama* OR Panamá* OR Paraguay* OR Paraguái* OR Peru* OR Perú* OR "Puerto Rico*" OR "Puerto Rican*" OR "Saint Kitts" OR "St Kitts" OR Nevis OR Kittitian* OR Nevisian* OR "Saint Lucia*" OR "St Lucia*" OR "Saint Vincent" OR "St Vincent" OR Vincentian* OR Grenadines OR "Sint Maarten*" OR "Saint Martin*" OR "St Martin*" OR "Saint Bart*" OR "St Bart*" OR Barthélemois OR "Saint Pierre" OR "St Pierre" OR Saint-Pierrais OR Pierrian* OR Miquelon* OR Surinam* OR Trinidad* OR Tobago* OR "Turks and Caicos" OR Uruguay* OR Venezuela* OR Caribbean OR "West Indies"))

AND PY 2000-2024

Limits: years limited to 2000-present, Academic Journals

**Results**: 26 results as of July 18, 2024. NB: Missing geographies added 0 additional results as of Aug. 26, 2024

**Global Index Medicus**

|  | Concept: Shigella | Concept: incidence/prevalence | Concept:  PAHO countries |
| --- | --- | --- | --- |
| Subject Descriptors | B03.440.450.425.850*  "Dysentery, Bacillary" | "Incidence"  "Prevalence"  "Cross-Sectional Studies"  E05.318.372.500.500*  Y09.010*  "Disease Hotspot" | "Latin America" Z01.107.169* Z01.107.757* "Mexico" Z01.107.084* Z01.639.040* "Caribbean People" M01.686.429* M01.686.685* "Netherlands Antilles" |
| Index |  |  | LILACS |
| Title, abstract, subject  (tw:()) or  Title, abstract, keyword  (ti:(), ab:(), kw:()) | Searched in title, abstract, and author keywords (:ti,ab,kw)  shigella* shigellosis "bacillary dysentery" "shiga bacillus dysentery" | Searched in title, abstract, and author keywords (:ti,ab,kw)  Incidence incidences prevalence prevalences cross-sectional **(association AND (study OR studies))**  "ecological studies" "ecological study" Transversal ((case OR cases) AND (control OR controls)) case-comparison case-compeer case-base case-referent "attributable fraction" "attributable fractions" "attributable risk" "attributable risks"    Searched in title only  Association, associations | Searched in title, abstract, author keywords, or journal title  **"Latin America" "Latin American" "Latin Americans" "Central America" "Central American" "Central Americans" "South America" "South American" "South Americans"** Anguilla* Antigua* Barbados Barbadian* Barbuda* Argentin* Aruba* Baham* Belize* Bolivia* Brazil* Brasil* "Virgin Island" "Virgin Islands" "Virgin Islander" "Virgin Islanders" "Cayman Island" "Cayman Islands" "Cayman Islander" "Cayman Islanders" Chile* Colombia* "Costa Rica" "Costa Rican" "Costa Ricans" Cuba* Curaçao* Dominica* Ecuador* "El Salvador" Salvadoran* "Falkland Island" "Falkland Islands" "Falkland Islander" "Falkland Islanders" Falklander* Grenada* Guadeloup* Guatemala Guatemalan* Guian* Guyan*Haiti* Haïti* Ayiti* Hispaniola Honduras Jamaica* Martinique Martiniquais* Martinican* Mexic* Méxic* Montserrat* "Netherlands Antilles" "Netherlands Antillean" "Netherlands Antilleans" Nicaragua* Panama* Panamá* Paraguay* Paraguái* Peru* Perú* "Puerto Rico" "Puerto Rican" "Puerto Ricans" "Saint Kitts" "St Kitts" Nevis Kittitian* Nevisian* "Saint Lucia" "Saint Lucian" "Saint Lucians" "St Lucia" "St Lucian" "St Lucians" "Saint Vincent" "St Vincent" Vincentian* Grenadines "Sint Maarten" "Sint Maartener" "Sint Maarteners" "Saint Martin" "Saint Martiner" "Saint Martiners" "Saint Martinois" "St Martin*" "St Martiner" "St Martiners" "St Martinois" "Saint Barthélemy" "Saint Barts" "St Barthélemy" "St Bart" Barthélemois "Saint Pierre" "St Pierre" Saint-Pierrais Pierrian* Miquelon* Surinam* Trinidad* Tobago* "Turks and Caicos" Uruguay* Venezuela*Caribbean "West Indies" |

(mh:(B03.440.450.425.850* OR "Dysentery, Bacillary") OR ti:(shigella* OR shigellosis OR "bacillary dysentery" OR "shiga bacillus dysentery") OR ab:( shigella* OR shigellosis OR "bacillary dysentery" OR "shiga bacillus dysentery") OR kw:(shigella* OR shigellosis OR "bacillary dysentery" OR "shiga bacillus dysentery"))

 AND

(mh:("Incidence" OR "Prevalence" OR "Cross-Sectional Studies" OR E05.318.372.500.500* OR Y09.010* OR "Disease Hotspot") OR ti:(incidence OR incidences OR prevalence OR prevalences OR cross-sectional OR **(association AND (study OR studies)) OR** "ecological studies" OR "ecological study" OR transversal OR ((case OR cases) AND (control OR controls)) OR case-comparison OR case-compeer OR case-base OR case-referent OR "attributable fraction" OR "attributable fractions" OR "attributable risk" OR "attributable risks") OR ab:( incidence OR incidences OR prevalence OR prevalences OR cross-sectional OR **(association AND (study OR studies)) OR** "ecological studies" OR "ecological study" OR transversal OR ((case OR cases) AND (control OR controls)) OR case-comparison OR case-compeer OR case-base OR case-referent OR "attributable fraction" OR "attributable fractions" OR "attributable risk" OR "attributable risks") OR kw:( incidence OR incidences OR prevalence OR prevalences OR cross-sectional OR **(association AND (study OR studies)) OR** "ecological studies" OR "ecological study" OR transversal OR ((case OR cases) AND (control OR controls)) OR case-comparison OR case-compeer OR case-base OR case-referent OR "attributable fraction" OR "attributable fractions" OR "attributable risk" OR "attributable risks") OR ti:(association OR associations))

AND

(( collection_gim:("LILACS")) OR mh:("Latin America" OR Z01.107.169* OR Z01.107.757* OR "Mexico" OR Z01.107.084* OR Z01.639.040* OR "Caribbean People" OR M01.686.429* OR M01.686.685* OR "Netherlands Antilles") OR ti:(**"Latin America" OR "Latin American" OR "Latin Americans" OR "Central America" OR "Central American" OR "Central Americans" OR "South America" OR "South American" OR "South Americans" OR** Anguilla* OR Antigua* OR Barbados OR Barbadian* OR Barbuda* OR Argentin* OR Aruba* OR Baham* OR Belize* OR Bolivia* OR Brazil* OR Brasil* OR "Virgin Island" OR "Virgin Islands" OR "Virgin Islander" OR "Virgin Islanders" OR "Cayman Island" OR "Cayman Islands" OR "Cayman Islander" OR "Cayman Islanders" OR Chile* OR Colombia* OR "Costa Rica" OR "Costa Rican" OR "Costa Ricans" OR Cuba* OR Curaçao* OR Dominica* OR Ecuador* OR "El Salvador" OR Salvadoran* OR "Falkland Island" OR "Falkland Islands" OR "Falkland Islander" OR "Falkland Islanders" OR Falklander* OR Grenada* OR Guadeloup* OR Guatemala OR Guatemalan* OR Guian* OR Guyan* OR Haiti* OR Haïti* OR Ayiti* OR Hispaniola OR Honduras OR Jamaica* OR Martinique OR Martiniquais* OR Martinican* OR Mexic* OR Méxic* OR Montserrat* OR "Netherlands Antilles" OR "Netherlands Antillean" OR "Netherlands Antilleans" OR Nicaragua* OR Panama* OR Panamá* OR Paraguay* OR Paraguái* OR Peru* OR Perú* OR "Puerto Rico" OR "Puerto Rican" OR "Puerto Ricans" OR "Saint Kitts" OR "St Kitts" OR Nevis OR Kittitian* OR Nevisian* OR "Saint Lucia" OR "Saint Lucian" OR "Saint Lucians" OR "St Lucia" OR "St Lucian" OR "St Lucians" OR "Saint Vincent" OR "St Vincent" OR Vincentian* OR Grenadines OR "Sint Maarten" OR "Sint Maartener" OR "Sint Maarteners" OR "Saint Martin" OR "Saint Martiner" OR "Saint Martiners" OR "Saint Martinois" OR "St Martin*" OR "St Martiner" OR "St Martiners" OR "St Martinois" OR "Saint Barthélemy" OR "Saint Barts" OR "St Barthélemy" OR "St Bart" OR Barthélemois OR "Saint Pierre" OR "St Pierre" OR Saint-Pierrais OR Pierrian* OR Miquelon* OR Surinam* OR Trinidad* OR Tobago* OR "Turks and Caicos" OR Uruguay* OR Venezuela* OR Caribbean OR "West Indies") OR ab:(**"Latin America" OR "Latin American" OR "Latin Americans" OR "Central America" OR "Central American" OR "Central Americans" OR "South America" OR "South American" OR "South Americans" OR** Anguilla* OR Antigua* OR Barbados OR Barbadian* OR Barbuda* OR Argentin* OR Aruba* OR Baham* OR Belize* OR Bolivia* OR Brazil* OR Brasil* OR "Virgin Island" OR "Virgin Islands" OR "Virgin Islander" OR "Virgin Islanders" OR "Cayman Island" OR "Cayman Islands" OR "Cayman Islander" OR "Cayman Islanders" OR Chile* OR Colombia* OR "Costa Rica" OR "Costa Rican" OR "Costa Ricans" OR Cuba* OR Curaçao* OR Dominica* OR Ecuador* OR "El Salvador" OR Salvadoran* OR "Falkland Island" OR "Falkland Islands" OR "Falkland Islander" OR "Falkland Islanders" OR Falklander* OR Grenada* OR Guadeloup* OR Guatemala OR Guatemalan* OR Guian* OR Guyan* OR Haiti* OR Haïti* OR Ayiti* OR Hispaniola OR Honduras OR Jamaica* OR Martinique OR Martiniquais* OR Martinican* OR Mexic* OR Méxic* OR Montserrat* OR "Netherlands Antilles" OR "Netherlands Antillean" OR "Netherlands Antilleans" OR Nicaragua* OR Panama* OR Panamá* OR Paraguay* OR Paraguái* OR Peru* OR Perú* OR "Puerto Rico" OR "Puerto Rican" OR "Puerto Ricans" OR "Saint Kitts" OR "St Kitts" OR Nevis OR Kittitian* OR Nevisian* OR "Saint Lucia" OR "Saint Lucian" OR "Saint Lucians" OR "St Lucia" OR "St Lucian" OR "St Lucians" OR "Saint Vincent" OR "St Vincent" OR Vincentian* OR Grenadines OR "Sint Maarten" OR "Sint Maartener" OR "Sint Maarteners" OR "Saint Martin" OR "Saint Martiner" OR "Saint Martiners" OR "Saint Martinois" OR "St Martin*" OR "St Martiner" OR "St Martiners" OR "St Martinois" OR "Saint Barthélemy" OR "Saint Barts" OR "St Barthélemy" OR "St Bart" OR Barthélemois OR "Saint Pierre" OR "St Pierre" OR Saint-Pierrais OR Pierrian* OR Miquelon* OR Surinam* OR Trinidad* OR Tobago* OR "Turks and Caicos" OR Uruguay* OR Venezuela* OR Caribbean OR "West Indies") OR kw:(**"Latin America" OR "Latin American" OR "Latin Americans" OR "Central America" OR "Central American" OR "Central Americans" OR "South America" OR "South American" OR "South Americans" OR** Anguilla* OR Antigua* OR Barbados OR Barbadian* OR Barbuda* OR Argentin* OR Aruba* OR Baham* OR Belize* OR Bolivia* OR Brazil* OR Brasil* OR "Virgin Island" OR "Virgin Islands" OR "Virgin Islander" OR "Virgin Islanders" OR "Cayman Island" OR "Cayman Islands" OR "Cayman Islander" OR "Cayman Islanders" OR Chile* OR Colombia* OR "Costa Rica" OR "Costa Rican" OR "Costa Ricans" OR Cuba* OR Curaçao* OR Dominica* OR Ecuador* OR "El Salvador" OR Salvadoran* OR "Falkland Island" OR "Falkland Islands" OR "Falkland Islander" OR "Falkland Islanders" OR Falklander* OR Grenada* OR Guadeloup* OR Guatemala OR Guatemalan* OR Guian* OR Guyan* OR Haiti* OR Haïti* OR Ayiti* OR Hispaniola OR Honduras OR Jamaica* OR Martinique OR Martiniquais* OR Martinican* OR Mexic* OR Méxic* OR Montserrat* OR "Netherlands Antilles" OR "Netherlands Antillean" OR "Netherlands Antilleans" OR Nicaragua* OR Panama* OR Panamá* OR Paraguay* OR Paraguái* OR Peru* OR Perú* OR "Puerto Rico" OR "Puerto Rican" OR "Puerto Ricans" OR "Saint Kitts" OR "St Kitts" OR Nevis OR Kittitian* OR Nevisian* OR "Saint Lucia" OR "Saint Lucian" OR "Saint Lucians" OR "St Lucia" OR "St Lucian" OR "St Lucians" OR "Saint Vincent" OR "St Vincent" OR Vincentian* OR Grenadines OR "Sint Maarten" OR "Sint Maartener" OR "Sint Maarteners" OR "Saint Martin" OR "Saint Martiner" OR "Saint Martiners" OR "Saint Martinois" OR "St Martin*" OR "St Martiner" OR "St Martiners" OR "St Martinois" OR "Saint Barthélemy" OR "Saint Barts" OR "St Barthélemy" OR "St Bart" OR Barthélemois OR "Saint Pierre" OR "St Pierre" OR Saint-Pierrais OR Pierrian* OR Miquelon* OR Surinam* OR Trinidad* OR Tobago* OR "Turks and Caicos" OR Uruguay* OR Venezuela* OR Caribbean OR "West Indies") OR ta:(**"Latin America" OR "Latin American" OR "Latin Americans" OR "Central America" OR "Central American" OR "Central Americans" OR "South America" OR "South American" OR "South Americans" OR** Anguilla* OR Antigua* OR Barbados OR Barbadian* OR Barbuda* OR Argentin* OR Aruba* OR Baham* OR Belize* OR Bolivia* OR Brazil* OR Brasil* OR "Virgin Island" OR "Virgin Islands" OR "Virgin Islander" OR "Virgin Islanders" OR "Cayman Island" OR "Cayman Islands" OR "Cayman Islander" OR "Cayman Islanders" OR Chile* OR Colombia* OR "Costa Rica" OR "Costa Rican" OR "Costa Ricans" OR Cuba* OR Curaçao* OR Dominica* OR Ecuador* OR "El Salvador" OR Salvadoran* OR "Falkland Island" OR "Falkland Islands" OR "Falkland Islander" OR "Falkland Islanders" OR Falklander* OR Grenada* OR Guadeloup* OR Guatemala OR Guatemalan* OR Guian* OR Guyan* OR Haiti* OR Haïti* OR Ayiti* OR Hispaniola OR Honduras OR Jamaica* OR Martinique OR Martiniquais* OR Martinican* OR Mexic* OR Méxic* OR Montserrat* OR "Netherlands Antilles" OR "Netherlands Antillean" OR "Netherlands Antilleans" OR Nicaragua* OR Panama* OR Panamá* OR Paraguay* OR Paraguái* OR Peru* OR Perú* OR "Puerto Rico" OR "Puerto Rican" OR "Puerto Ricans" OR "Saint Kitts" OR "St Kitts" OR Nevis OR Kittitian* OR Nevisian* OR "Saint Lucia" OR "Saint Lucian" OR "Saint Lucians" OR "St Lucia" OR "St Lucian" OR "St Lucians" OR "Saint Vincent" OR "St Vincent" OR Vincentian* OR Grenadines OR "Sint Maarten" OR "Sint Maartener" OR "Sint Maarteners" OR "Saint Martin" OR "Saint Martiner" OR "Saint Martiners" OR "Saint Martinois" OR "St Martin*" OR "St Martiner" OR "St Martiners" OR "St Martinois" OR "Saint Barthélemy" OR "Saint Barts" OR "St Barthélemy" OR "St Bart" OR Barthélemois OR "Saint Pierre" OR "St Pierre" OR Saint-Pierrais OR Pierrian* OR Miquelon* OR Surinam* OR Trinidad* OR Tobago* OR "Turks and Caicos" OR Uruguay* OR Venezuela* OR Caribbean OR "West Indies"))

AND (year_cluster:[2000 TO 2024])

Limits: years limited to 2000-present

**Results**: 73 results as of July 18, 2024

*Missing geographies added 0 additional results as of Aug. 26, 2024*

Web of Science – SCI-EXPANDED, SSCI, AHCI, ESCI

|  | Concept: Shigella | Concept: incidence/prevalence | Concept: PAHO Countries |
| --- | --- | --- | --- |
| Topic  (searches title, abstract, author keywords, Keywords Plus®) | Searched in Topic  shigella*  shigellosis  "bacillary dysentery"  "shiga bacillus dysentery" | Searched in Topic  incidence incidences prevalence prevalences cross-sectional **(association NEAR/2 (study OR studies))** "ecological studies" "ecological study" Transversal ((case OR cases) AND (control OR controls)) case-comparison case-compeer case-base case-referent "attributable fraction*" "attributable risk*"    Searched in title only  Association associations | Searched in Topic and journal title  **"Latin America*" "Central America*" "South America*"** Anguilla* Antigua* Barbados Barbadian* Barbuda* Argentin* Aruba* Baham* Belize* Bolivia* Brazil* Brasil* "Virgin Island*" "Cayman Island*" Chile* Colombia* "Costa Rica*" Cuba* Curaçao* Dominica* Ecuador* "El Salvador" Salvadoran* "Falkland Island*" Falklander* Grenada* Guadeloup* Guatemala Guatemalan* Guian* Guyan* Haiti* Haïti* Ayiti* Hispaniola Hondura* Jamaica* Martinique Martiniquais* Martinican* Mexic* Méxic* Montserrat* "Netherlands Antille*" Nicaragua* Panama* Panamá* Paraguay* Paraguái* Peru* Perú* "Puerto Rico*" "Puerto Rican*" "Saint Kitts" "St Kitts" Nevis Kittitian* Nevisian* "Saint Lucia*" "St Lucia*" "Saint Vincent" "St Vincent" Vincentian* Grenadines "Sint Maarten*" "Saint Martin*" "St Martin*" "Saint Bart*" "St Bart*" Barthélemois "Saint Pierre" "St Pierre" Saint-Pierrais Pierrian* Miquelon* Surinam* Trinidad* Tobago* "Turks and Caicos" Uruguay* Venezuela* Caribbean "West Indies" |

Running note: Run string from the Advanced Search. Set date limits on Advanced Search page or in the results sidebar.

TS=(shigella* OR shigellosis OR "bacillary dysentery" OR "shiga bacillus dysentery")

 AND

( TS=(incidence OR incidences OR prevalence OR prevalences OR cross-sectional OR **(association NEAR/2 (study OR studies)) OR** "ecological studies" OR "ecological study" OR transversal OR ((case OR cases) AND (control OR controls)) OR case-comparison OR case-compeer OR case-base OR case-referent OR "attributable fraction*" OR "attributable risk*") OR TI=(association OR associations) )

AND

(TS=(**"Latin America*" OR "Central America*" OR "South America*" OR** Anguilla* OR Antigua* OR Barbados OR Barbadian* OR Barbuda* OR Argentin* OR Aruba* OR Baham* OR Belize* OR Bolivia* OR Brazil* OR Brasil* OR "Virgin Island*" OR "Cayman Island*" OR Chile* OR Colombia* OR "Costa Rica*" OR Cuba* OR Curaçao* OR Dominica* OR Ecuador* OR "El Salvador" OR Salvadoran* OR "Falkland Island*" OR Falklander* OR Grenada* OR Guadeloup* OR Guatemala OR Guatemalan* OR Guian* OR Guyan* OR Haiti* OR Haïti* OR Ayiti* OR Hispaniola OR Hondura* OR Jamaica* OR Martinique OR Martiniquais* OR Martinican* OR Mexic* OR Méxic* OR Montserrat* OR "Netherlands Antille*" OR Nicaragua* OR Panama* OR Panamá* OR Paraguay* OR Paraguái* OR Peru* OR Perú* OR "Puerto Rico*" OR "Puerto Rican*" OR "Saint Kitts" OR "St Kitts" OR Nevis OR Kittitian* OR Nevisian* OR "Saint Lucia*" OR "St Lucia*" OR "Saint Vincent" OR "St Vincent" OR Vincentian* OR Grenadines OR "Sint Maarten*" OR "Saint Martin*" OR "St Martin*" OR "Saint Bart*" OR "St Bart*" OR Barthélemois OR "Saint Pierre" OR "St Pierre" OR Saint-Pierrais OR Pierrian* OR Miquelon* OR Surinam* OR Trinidad* OR Tobago* OR "Turks and Caicos" OR Uruguay* OR Venezuela* OR Caribbean OR "West Indies"))

Limits: years limited to 2000-present

**Results**: 181 results as of July 18, 2024; NB: *Missing geographies added 3 additional results as of Aug. 26, 20*
