# Supplementary material for: Burden of Shigella among children with diarrhea in the Americas: A systematic review and meta-analysis
Source: PLoS Negl Trop Dis. 2025 Aug 18;19(8):e0013393. doi: 10.1371/journal.pntd.0013393 (PMC12413091; doi:10.1371/journal.pntd.0013393)
Supplement: S3 Table — (DOCX) [file pntd.0013393.s005.docx]

**S3 Table: Adapted Joanna Briggs Institute (JBI) Quality Assessment Tool**

| **Criteria** | **Description** | **Score** |
| --- | --- | --- |
| Inclusion criteria defined | The authors should provide clear inclusion and exclusion criteria that they developed prior to recruitment of the study participants. The inclusion/exclusion criteria should be specified (e.g., risk, stage of disease progression) with sufficient detail and all the necessary information critical to the study | Completely satisfied |
|  |  | Partially satisfied |
|  |  | Not satisfied |
|  |  | NA |
| Study subjects and setting described | The study sample should be described in sufficient detail so that other researchers can determine if it is comparable to the population of interest to them. The authors should provide a clear description of the population from which the study participants were selected or recruited, including demographics, location, and time period | Completely satisfied |
|  |  | Partially satisfied |
|  |  | Not satisfied |
|  |  | NA |
| Sample size | Regarding participants <72 months with diarrhea: >50 completely satisfied; 10-50 partially satisfied; and <10 not satisfied | Completely satisfied |
|  |  | Partially satisfied |
|  |  | Not satisfied |
|  |  | NA |
| Type of diarrhea defined | Complete responses include duration and/or definition of severity indicators (e.g. dehydration, blood in stool) | Completely satisfied |
|  |  | Partially satisfied |
|  |  | Not satisfied |
|  |  | NA |
| Shigella confirmation described | Lab method and description of media, platform, probe, or gene completely satisfies criteria. Only e.g. "confirmed with PCR" is a partial description | Completely satisfied |
|  |  | Partially satisfied |
|  |  | Not satisfied |
|  |  | NA |
| Metrics reported with confidence intervals | Completely satisfied if our metric of interest has CIs. Partially satisfied if any metric in paper has CIs | Completely satisfied |
|  |  | Partially satisfied |
|  |  | Not satisfied |
|  |  | NA |
