# Supplementary material for: Burden of Shigella among children with diarrhea in the Americas: A systematic review and meta-analysis
Source: PLoS Negl Trop Dis. 2025 Aug 18;19(8):e0013393. doi: 10.1371/journal.pntd.0013393 (PMC12413091; doi:10.1371/journal.pntd.0013393)
Supplement: S4 Table — (DOCX) [file pntd.0013393.s006.docx]

**S4 Table: Burden Estimates from Study Sites in the Amazon Region**


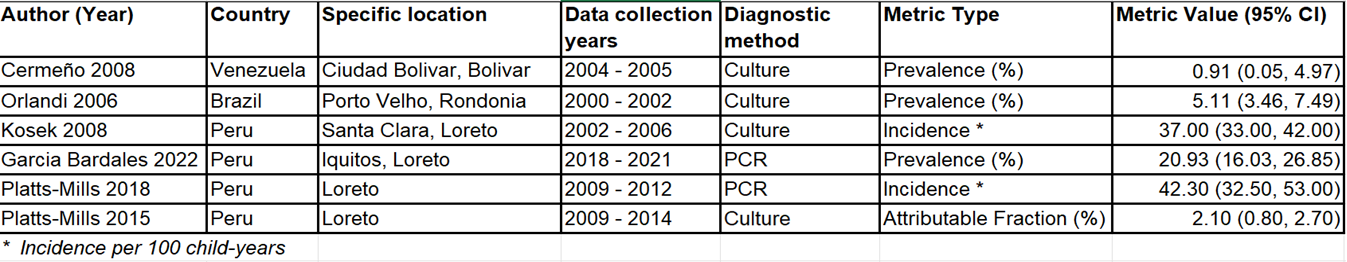


**References**

1. Cermeño JR, Hernández de Cuesta I, Camaripano M, Medina N, Guevara A, Hernández Rivero C. Etiología de diarrea aguda en niños menores de 5 años Ciudad Bolívar, Venezuela. Revista de la Sociedad Venezolana de Microbiología. 2008;28: 55–60.
2. Orlandi PP, Magalhães GF, Matos NB, Silva T, Penatti M, Nogueira PA, et al. Etiology of diarrheal infections in children of Porto Velho (Rondonia, Western Amazon region, Brazil). Braz J Med Biol Res. 2006;39: 507–517. doi:10.1590/S0100-879X2006000400011
3. Kosek M, Yori PP, Pan WK, Olortegui MP, Gilman RH, Perez J, et al. Epidemiology of Highly Endemic Multiply Antibiotic-Resistant Shigellosis in Children in the Peruvian Amazon. Pediatrics. 2008;122: e541–e549. doi:10.1542/peds.2008-0458
4. Garcia Bardales PF, Schiaffino F, Huynh S, Paredes Olortegui M, Peñataro Yori P, Pinedo Vasquez T, et al. “Candidatus Campylobacter infans” detection is not associated with diarrhea in children under the age of 2 in Peru. Senok A, editor. PLoS Negl Trop Dis. 2022;16: e0010869. doi:10.1371/journal.pntd.0010869
5. Platts-Mills JA, Liu J, Rogawski ET, Kabir F, Lertsethtakarn P, Siguas M, et al. Use of quantitative molecular diagnostic methods to assess the aetiology, burden, and clinical characteristics of diarrhoea in children in low-resource settings: a reanalysis of the MAL-ED cohort study. The Lancet Global Health. 2018;6: e1309–e1318. doi:10.1016/S2214-109X(18)30349-8
6. Platts-Mills JA, Babji S, Bodhidatta L, Gratz J, Haque R, Havt A, et al. Pathogen-specific burdens of community diarrhoea in developing countries: a multisite birth cohort study (MAL-ED). The Lancet Global Health. 2015;3: e564–e575. doi:10.1016/S2214-109X(15)00151-5
